# Liquid–Liquid Phase Separation‐Mediated Photocatalytic Subcellular Hybrid System for Highly Efficient Hydrogen Production

**DOI:** 10.1002/advs.202400097

**Published:** 2024-04-04

**Authors:** Xiaoxuan Yu, Hui Li, Chengchen Xu, Zhengwei Xu, Shuheng Chen, Wang Liu, Tianlong Zhang, Hongcheng Sun, Yan Ge, Zhenhui Qi, Junqiu Liu

**Affiliations:** ^1^ Key Laboratory of Organosilicon Chemistry and Material Technology Ministry of Education College of Material Chemistry and Chemical Engineering Hangzhou Normal University Hangzhou 311121 China; ^2^ Sino‐German Joint Research Lab for Space Biomaterials and Translational Technology School of Life Sciences Northwestern Polytechnical University Xi'an 710072 China

**Keywords:** biohybrid, compartmentalization, hydrogen production, membranous organelle, photocatalysis

## Abstract

Plant chloroplasts have a highly compartmentalized interior, essential for executing photocatalytic functions. However, the construction of a photocatalytic reaction compartment similar to chloroplasts in inorganic–biological hybrid systems (IBS) has not been reported. Drawing inspiration from the compartmentalized chloroplast and the phenomenon of liquid–liquid phase separation, herein, a new strategy is first developed for constructing a photocatalytic subcellular hybrid system through liquid–liquid phase separation technology in living cells. Photosensitizers and in vivo expressed hydrogenases are designed to coassemble within the cell to create subcellular compartments for synergetic photocatalysis. This compartmentalization facilitates efficient electron transfer and light energy utilization, resulting in highly effective H_2_ production. The subcellular compartments hybrid system (HM/IBSCS) exhibits a nearly 87‐fold increase in H_2_ production compared to the bare bacteria/hybrid system. Furthermore, the intracellular compartments of the photocatalytic reactor enhance the system's stability obviously, with the bacteria maintaining approximately 81% of their H_2_ production activity even after undergoing five cycles of photocatalytic hydrogen production. The research brings forward visionary prospects for the field of semi‐artificial photosynthesis, offering new possibilities for advancements in areas such as renewable energy, biomanufacturing, and genetic engineering.

## Graphical abstract

Constructing membraneless organelles in living bacteria for semiartificial photosynthesis is a pioneering approach. This innovative strategy involves precise assembly of specific proteins, promoting quantum dots and hydrogenases, providing a promising approach to improving the efficiency and stability of semiartificial photosynthetic systems.

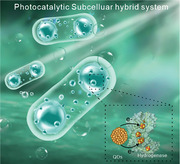

## 1 Introduction

Under the current energy challenges, the excessive use of fossil fuels has led to severe environmental issues,^[^ ^1^, ^2^ ^]^ including greenhouse gas emissions, climate change, acid rain, and ozone layer depletion, posing a significant threat to the global economy and societal development.^[^ ^3^, ^4^ ^]^ Therefore, there is an urgent need to transition towards renewable and clean energy sources.^[^ ^5^, ^6^ ^]^ To address this demand, harnessing renewable energy such as solar power for sustainable chemical production,^[^ ^7^, ^8^ ^]^ including hydrogen and other fuels has become imperative.^[^ ^9^ ^]^ The semi‐artificial photosynthetic hydrogen production system has made remarkable strides in this context, involving the integration of bacteria with semiconductors, categorized into extracellular, cytoplasmic, and periplasmic photosensitive biohybrid systems.^[^ ^10^, ^11^, ^12^, ^13^ ^]^

**Figure 1:**
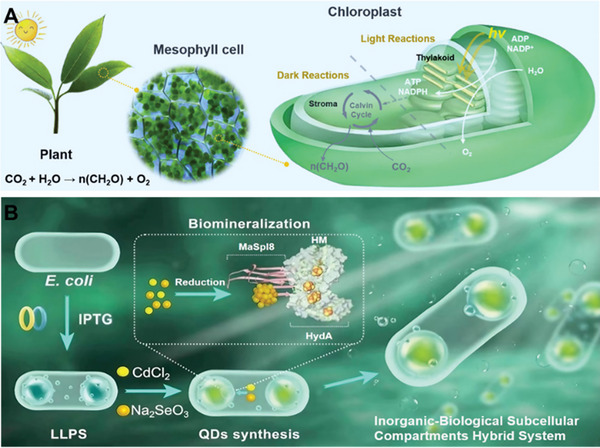
A) Plants possess the capability to reduce CO_2_ into carbohydrates due to the presence of chloroplasts in mesophyll cells of plant leaves, where photosynthesis occurs. Subcellular organelles called thylakoids, located within chloroplasts, facilitate the light reactions. The photosynthetic membranes on thylakoids capture light energy and channel electrons, ultimately generating ATP and NADPH. Dark reactions take place in the chloroplast stroma, utilizing the ATP and NADPH generated from light reactions to reduce CO_2_ into carbohydrates (*n*(CH_2_O)). Thylakoids confine high‐energy photochemical reactions within specific compartments, preventing the escape of high‐energy electrons. B) Schematic diagram of constructing an inorganic‐biological subcellular compartments hybrid system in *E. coli* for solar hydrogen production.

However, these systems face challenges, as illustrated in Figure S1 (Supporting Information): 1) Restricted material exchange may lead to reduced reaction efficiency;^[^ ^14^ ^]^ 2) Uncertainty in the binding of enzyme/photosensitizer mixtures may affect reaction selectivity;^[^ ^15^, ^16^ ^]^ 3) Unclear reaction regions may result in decreased specificity in product formation;^[^ ^17^ ^]^ 4) Potential cellular toxicity may trigger bacterial stress responses.^[^ ^17^ ^]^ The reason why natural photosynthesis can successfully address the challenges faced by the semi‐artificial photosynthetic hydrogen production system mentioned above is due to its ingenious compartmentalization mechanism, which isolates different stages of photosynthesis (**Figure** **1**A).^[^ ^18^, ^19^, ^20^, ^21^, ^22^ ^]^ This enables the natural photosynthetic system to efficiently separate various stages of photosynthesis during the process of converting radiant energy into chemical potential.^[^ ^23^, ^24^ ^]^ It forms an effective compartmentalization mechanism inside the cell, enhancing energy capture efficiency and acting as a barrier to prevent harmful reactions and the diffusion of harmful substances to other cellular regions.^[^ ^25^, ^26^ ^]^

In contrast to the chloroplast membrane compartmentalization mechanism, another widely accepted compartmentalization phenomenon exists in cells, known as liquid‐liquid phase separation (LLPS).^[^ ^27^, ^28^, ^29^ ^]^ It involves the precise and ordered aggregation of intrinsically disordered proteins in designated areas, forming membraneless organelles (MLO).^[^ ^30^, ^31^ ^]^ For instance, Liu et al. reported the construction of artificial MLO in brewer's yeast to enhance chemical production.^[^ ^32^ ^]^ Xia et al. engineered phase‐separated silk protein condensates in *Escherichia coli* (*E. coli)*, effectively enriching catalytic enzymes and advancing the functionality of directed biosynthesis of quantum dots (QDs).^[^ ^33^, ^34^ ^]^ Wang et al. employed LLPS in *E. coli* to achieve the compartmentalized synthesis of α‐farnesene.^[^ ^28^ ^]^ Therefore, there is the potential to provide a promising approach for semi‐artificial photosynthetic systems.

**Figure 2:**
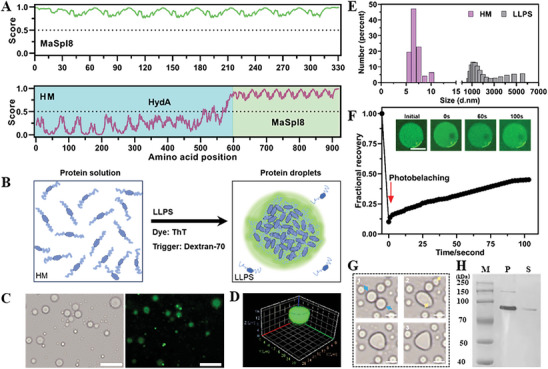
In vitro phase separation system based on HM fusion protein. Prediction of protein disorder of A,upper) MaSpI8, a fusion protein of MaSpI8, and hydrogenase (HM, bottom) using the IUPRed2 program. A score > 0.5 is considered to have phase separation capability. B) The hypothetical schematic diagram illustrates the fusion expression structure of HM, which allows HM to form compartmentalized condensates via inter‐chain interactions of the β‐sheets when mixed with the macromolecular crowding agent dextran‐70. C) The confocal laser scanning microscopy images of in vitro reconstituted ThT‐stained HM condensates. Scale bar = 10 µm. D) 3D confocal scanning image of HM condensate. (E) When mixed with the macroscopic crowding agent dextran 70, the particle size of the HM solution increases from small to large. (F) Representative fluorescence recovery of a partial photo‐bleached droplet. The inset shows images of photobleaching on the droplet. Scale bars = 5 µm. G) Phase formation under light microscopy images showing the fusion of three individual liquid droplets. H) SDS‐PAGE analysis revealed the distribution of proteins in the phase separation system. The HM is predominantly found in the condensed phase/pellet (*P*), while only a small amount was detected in the diluted phase/supernatant (*S*).

Inspired by chloroplast photosynthesis and LLPS leading to compartmentalization, herein, we have developed a new strategy for semiartificial photosynthesis hydrogen production through utilizing non‐native photosynthetic bacteria (*E. coli*) as the chassis and employing genetic engineering techniques to create subcellular compartments. The approach involves the formation of an inorganic‐biological subcellular compartments hybrid system (IBSCS) by coassembly of recombinant hydrogenase (HydA) and silk protein (MaSpI8/IDPs) fused protein (HM) with QDs (Figure 1B). Specifically, MaSpI8/IDPs were employed as a phase‐separation inducer (**Figure** **2**A), prompting bacteria to form subcellular compartments.^[^ ^33^, ^34^ ^]^ Moreover, MaSpI8/IDPs possess a unique amino acid sequence that allows for targeted synthesis of biocompatible QDs. In the end, we successfully established subcellular compartments within live cells, creating distinct regions for high‐energy photocatalytic reactions. The subcellular compartments formed by HM fusion proteins promote an increase in local hydrogenase concentration in *E. coli*. The spatial confinement of hydrogenase and QDs facilitates efficient electron transfer and maximizes the utilization of light energy, thereby achieving effective hydrogen production in IBSCS. Simultaneously, high‐energy electron holes are confined within subcellular compartments, preventing the overflow of reactive oxygen species (ROS), and thus enhancing the stability of the system. This method extends the frontier of semi‐artificial photosynthesis, offering fresh perspectives for the development of sustainable future energy solutions.

## 2 Results and Discussion

### 2.1 In Vitro and In Vivo Construction of Subcellular Compartments

Soluble spider silk proteins undergo LLPS to form the primary fibers of spider silk, which are enriched with residues of proline, glycine, and serine.^[^ ^35^, ^36^, ^37^ ^]^ It has been shown that the engineered MaSpI8/IDPs derived from spider silk proteins possess the capacity to create membrane less compartments within *E. coli*, which might be a preferred solution for constructing IBSCS.^[^ ^33^, ^34^ ^]^ Herein, the IUPRed2 program was employed to theoretically simulate the designed protein sequence,^[^ ^38^ ^]^ demonstrating that the fusion of hydrogenase with MaSpI8/IDPs theoretically possesses the capability for LLPS (Figure 2A and Figure S2, Supporting Information). Engineered plasmids were successfully constructed for the expression of both hydrogenase and its fusion with MaSpl8/IDPs using a dual plasmid expression system (Figure S3 and Table S1, Supporting Information). After being transformed into *E. coli* BL21 (DE3) cells (Figure S3 and Tables S1 and S2, Supporting Information), the engineered bacteria were grown at 37 °C in LB media supplemented with appropriate antibiotics to reach OD600 ≈0.6 before 0.1 × 10^−3^ m IPTG was added to induce protein expression. The successful expression of the HM fusion protein was confirmed by SDS‐PAGE analysis (Figure S4A,B, Supporting Information), and it demonstrated hydrogen production activity in *E. coli* cells (Figure S5A, Supporting Information), and no significant difference was found in hydrogenase activity across different engineered strains. By monitoring cell growth, we found that the expression of HydA and HM did not have any adverse effects on cell growth, indicating the biocompatibility of proteins with *E. coli* (Figure S5B, Supporting Information). The purified and overexpressed MaSpI8/IDPs fusion protein in *E. coli* can spontaneously form liquid condensates,^[^ ^33^, ^34^ ^]^ but it is unknown whether HydA‐MaSpI8 fusion protein (HM) can form a condensate. Next, we studied whether the MaSpI8 protein would support compartmentalized HM formation in vivo and in vitro.

LLPS is the foundation for the formation of membraneless compartments in cells.^[^ ^39^, ^40^, ^41^ ^]^ dextran‐70 was utilized to mimic the physiologically crowded environment inside cells and attempted to generate condensates of the HM fusion protein in vitro (Figure 2B and Figure S5C, Supporting Information). Upon adding the HM solution, the dextran‐70 immediately turned turbid,^[^ ^41^ ^]^ which indicates the formation of micron‐sized droplets through LLPS (Figure 2C,D). Dynamic light scattering (DLS) results revealed the hydrodynamic size was dramatically increased from ≈5 nm to above 1 µm after introducing dextran 70 into the solution (Figure 2E). The fluorescence recovery method (FRAP) after photobleaching was applied to analyze the dynamics inside the droplets, a particular condensate droplet underwent partial photobleaching. Within 100 s, the fluorescence signal at the photobleached region showed a recovery of approximately 40%, indicating the fluidic nature of the condensate (Figure 2F). Due to the fluidic nature of microdroplets, smaller droplets typically tended to fuse, forming larger droplets (Figure 2G). The β‐sheet‐rich HM protein,^[^ ^42^, ^43^ ^]^ after staining with thioflavin T (ThT), exhibited green fluorescence within the microdroplets (Figure 2C,D), indicating that the main component involved in the formation of LLPS was the HM protein. The HM fusion protein condensates in the solutions can be precipitated by centrifugation to separate the two phases, the supernatant/diluted phase (S phase) and the precipitate/condensed phase (P phase). Precipitant was quantified by SDS‐PAGE and most of the HM proteins were found in the P phase, indicating a strong tendency of LLPS (Figure 2H). We can speculate that in the case of HM protein forming subcellular compartments, the concentration of hydrogenase locally increases, which will facilitate the reaction. The liquid droplets formed through LLPS are highly dynamic and allow for the exchange of internal and external substances.^[^ ^36^, ^44^ ^]^ Therefore, fusing MaSpI8/IDPs at the C‐terminus of HydA can be expressed in *E. coli* and maintain self‐assembled LLPS characteristics in vitro.

**Figure 3:**
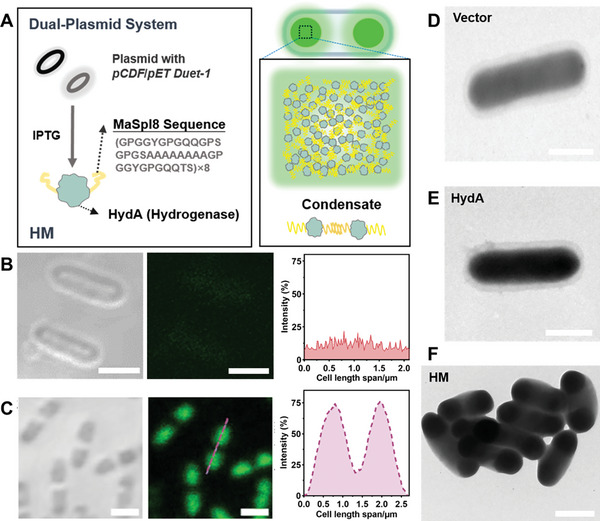
The CLSM and TEM images of different *E. coli* cells at 24 h after 0.1 × 10^−3^ m IPTG induction. A) Schematic diagram of the dual‐plasmid expression system for the HM fusion protein. By fusing MaSpI8 at the C‐terminus of HydA, utilizing the interactions of inter‐chain β‐folding, it facilitates the formation of condensates at the poles of bacteria. These liquid‐liquid phase‐separated membraneless organelles exhibit high dynamism. B) Bright‐field and CLSM images of *E. coli* HM protein‐expressing cells stained with ThT along with C) fluorescence intensity line‐cuts. TEM images of the expression of D) Vector, E) HydA, or F) HM in *E. coil* that have been immobilized using paraformaldehyde. Notably, the images reveal that bacteria expressing HydA or HM proteins exhibited deeper shades, with the HM group displaying intensified coloration predominantly localized at the bacterial poles. Scale bars = 1 µm.

To verify whether HM protein supports the formation of condensates within living *E. coli* cells, a confocal laser scanning microscope (CLSM) was used to observe the subcellular compartments in living *E. coli* cells. As shown in **Figure** **3**A, it was revealed the expression of the HM condensates spatially localized near the pole regions of cells (Figure 3C), while these condensates were not observed in the control cells (Figure 3B and Figure S6, Supporting Information), which indicated the HM proteins can form protein condensates in the cellular environment. The overexpression of this protein mildly affected bacterial morphology to some extent, but this does not hinder bacterial growth. Meanwhile, transmission electron microscope (TEM) images demonstrated that the expression of HM resulted in dark shadows at the poles of the recombinant *E. coli*, which may be due to protein enrichment, indicating the presence of subcellular compartments (Figure 3F). The expression of HydA in bacteria presents diffuse cytoplasmic shadows (Figure 3E). No shaded areas were observed in the vector bacteria (Figure 3D). The LLPS mediated by MaSpI8/IDPs based on spider silk protein is mainly based on its rich proline, glycine, low complexity, and modular sequence, driven by weak multivalent interactions, including electrostatic interactions, cation–π, π–π, and other hydrophobic contacts.^[^ ^45^, ^46^, ^47^ ^]^ The formation of LLPS/MLO within *E. coli* cells helps to isolate and protect exogenous proteins,^[^ ^48^, ^49^ ^]^ resist protease hydrolysis, and extend the half‐life enzyme.

### 2.2 Construction of Inorganic‐Biological Subcellular Compartments Hybrid System

**Figure 4:**
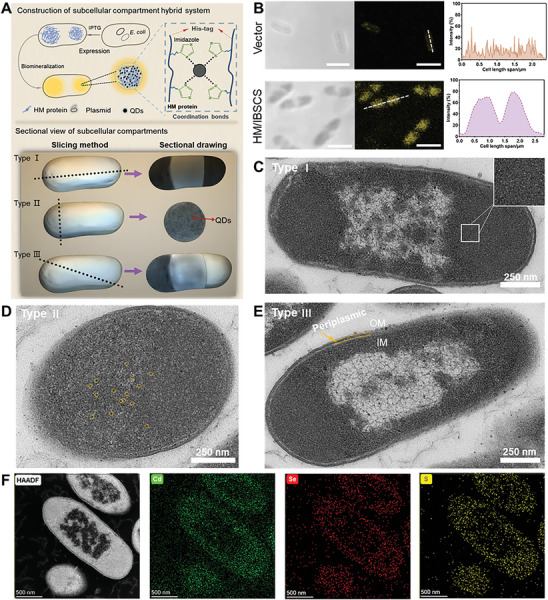
Formation of subcellular compartments hybrid system in *E. coli*. A) Schematic diagram of the construct biocompatible CdSe*_x_* S_1‐_ *_x_* QDs in *E. coli* expressing HM proteins (top) and Sectional view of subcellular compartments (bottom). B) The CLSM images of CdSe*_x_* S_1‐_ *_x_* QDs mineralized in different hybrid systems showed distinct undulating fluorescence peaks in the fluorescence intensity line‐cuts analysis. Bio‐TEM imaging of subcellular compartments using three different slicing methods. C) Type I: *E. coli* is divided into two equal parts with subcellular compartments located at opposite poles of the bacterium. D) Type II: The subcellular compartments are bisected. The cross‐section displays subcellular compartments and internal black dots as QDs (orange circles). E) Type III: Subcellular compartments are partially severed (darker region), Om: out membrane, Im: inner membrane. F) Bio‐TEM image of thin‐sectioned HM/IBSCS and the corresponding EDS mapping of Cd, S, and Se element.

Biomineralization involves the synthesis of endogenous enzymes under environmentally friendly conditions, imparting biostability and biocompatibility.^[^ ^50^, ^51^, ^52^ ^]^ CdSe_x_S_1‐_ *_x_* QDs are commonly used as an excellent model for intracellular mineralization in *E. coli*.^[^ ^33^, ^53^, ^54^ ^]^ The HM‐expressing *E. coli* cells were exposed to CdCl_2_, Na_2_SeO_3,_ and cysteine (Cys) to intracellular biomineralization QDs (**Figure** **4**A). The CLSM imaging revealed the formation of fluorescent QDs that were spatially localized near the pole regions of HM‐expressing cells (Figure 4B), whereas the control cells rarely supported the biogenesis of fluorescent QDs, and the fluorescence was diffuse (Figure S7, Supporting Information). All cells underwent uniform treatment conditions, the factor of protein misfolding and enrichment in *E. coli* caused by metal ions inducing bacterial oxidative stress was also ruled out.^[^ ^55^, ^56^ ^]^ Under bright‐field conditions, it was observed that the protein condensates compartmentalized the cytosolic of *E. coli* and exhibited localization with the fluorescence of QDs. Next, we used TEM to demonstrate from multiple perspectives that QDs synthesized in living cells spontaneously assemble with HM proteins at the two poles of bacteria to form subcellular compartments. Bio‐TEM and energy‐dispersive X‐ray spectroscopy (EDS) mapping were carried out to verify the location of the QDs in bacterial cytoplasm. Bio‐TEM imaging of subcellular compartments using three different slicing methods showed a significant accumulation of protein condensates at the two poles of bacteria and a dense distribution of black QDs in the middle (Figure 4 and Figure S8, Supporting Information). The Cd, Se, and S elements exhibited a bipolar distribution of protein condensates in *E. coli* (Figure 4F). Moreover, the three elemental distributions of the entire bacteria were almost consistent, indicating a precise spatial overlap between QDs and the HM condensate inside the bacteria. The quantification of the atomic fraction indicated that the Cd, Se, and S elements in the condensate are significantly higher than those in the bulk phase (Figure S8, Supporting Information). These results further demonstrate that QDs coassemble with HM to form subcellular compartments. On the contrary, the control cells exhibited limited fluorescence within living cells, which can be attributed to the fluorescence quenching caused by the aggregation of QDS without MaSpI8 template restriction (Figures S9 and S14, Supporting Information).^[^ ^33^ ^]^ The TEM results also confirmed large particulate precipitates in *E. coli* and dispersed element distribution in the control group (Figure S9B, Supporting Information). These results indicated that HM protein forms protein condensates/MLO in *E. coli* cells and can promote in situ synthesis of bio‐QDs, further forming subcellular compartments where HM protein condensates and QDs coassemble.

The X‐ray photoelectron spectroscopy (XPS) results provided valuable insights into the elemental composition of the HM/IBSCS. The XPS data analysis revealed distinct features associated with the presence of Cd, Se, and S elements (Figure S10, Supporting Information). The Cd3d spectrum displayed characteristic peaks at 405.29 and 412.09 eV.^[^ ^57^ ^]^ corresponding to Cd3d_5/2_ and Cd3d_3/2_, respectively. Similarly, the XPS spectrum involved Se3d peaks and the Se element exhibited S2p peaks.^[^ ^58^, ^59^ ^]^ These XPS results confirmed the presence of cadmium, selenium, and sulfur elements within the HM/IBSCS. Furthermore, the concentration of Cd elements in the solution of the biohybrid system was further measured using inductively coupled plasma‐atomic emission spectroscopy (ICP‐AES). Within the same timeframe, when compared to the HydA IBS system, the HM/IBSCS exhibited a higher concentration of Cd ions (Figure S10F, Supporting Information). This indicates that the internal subcellular compartments enable *E. coli* to uptake a higher amount of Cd ions. Further, overexpression of this protein can mitigate the impact of Cd^2+^ on bacterial activity, based on MTT activity detection (Figure S11, Supporting Information). There was no significant toxicity to cells during the biosynthesis of QDs within the cell. Based on the above results, HM fusing protein can be expressed in *E. coli* maintain self‐assembled LLPS characteristics in vivo, and improve the tolerance of *E. coli* to Cd ions. This could be attributed to the compartmentalization effect of the condensates, which immobilizes Cd^2+^ at the poles of bacteria, thereby reducing its impact on other cellular processes.

Notably, bacterial proliferation and cell division result in a small number of unipolar subcellular compartments in *E. coli* after QDs mineralization (Figure S12, Supporting Information). The subcellular compartments in living *E. coli* were examined using CLSM. It was observed that the yellow fluorescence of QDs was dispersed in bipolar or unipolar *E. coli* and this speculation was supported by bio‐TEM of thin‐sectioned and EDS mapping (Figure S13, Supporting Information). Cd, Se, and S elements exhibit spatially oriented distribution, with aggregated distribution at the single poles of *E. coli* (Figure S13D, Supporting Information). During the process of inducing protein expression in bacteria, it was usually considered that bacteria stop growing. When the growth of bacteria was restored. These protein/QDs aggregates were retained to form a unipolar form during bacterial division. This also indicates that bacterial proliferation and division do not affect the state of photocatalytic subcellular organelles.

To investigate the specific affinity of HM fusion proteins for QDs, we conducted a detailed study on the protein sequence. According to previous reports,^[^ ^33^ ^]^ the protein sequence of HM contains two sets of His‐tags bound to the MaSpI8/IDPs protein (Table S2, Supporting Information), which may be due to the purification tags inherent in the plasmid vector. Histidine residues can form strong metal coordination bonds with Cd, utilizing the imidazole group effectively capturing and immobilizing QDs.^[^ ^60^, ^61^ ^]^ This suggests that the interaction between QDs and HM was facilitated by the presence of His‐tags in the protein sequence. We reconstructed a strain expressing hydrogenase fusion of two sets of His‐tags (HH, without MaSpI8/IDPs) to investigate whether subcellular compartment construction can be achieved in the presence of two groups of His‐tag (Figure S14, Supporting Information). The theoretical prediction results show that the HH protein sequence has a score of less than 0.5 and does not possess the characteristics of LLPS (Figure S14A, Supporting Information). Additional protein purification was undertaken to create in vitro droplets based on the HH protein. However, microscopic examination revealed the absence of noticeable droplets, and DLS data indicated a minimal change in HH protein solution size post‐addition of dextran‐70 (Figure S14B,C, Supporting Information). While stained and QDs mineralized *E. coli* expressing HH protein exhibited restricted fluorescence, no bipolar aggregation was noted. No aggregation distribution was observed in the ThT/QDs fluorescence of living cells (Figure S14D,E, Supporting Information). These results underscored the essential role of MaSpI8/IDPs in the formation of subcellular compartments within live *E. coli*. These results indicate that overexpression of HM protein in *E. coli* promotes the formation of protein condensates, establishing membraneless subcellular compartments in the cytoplasm of bacteria, thereby achieving co‐assembly of subcellular compartments and QDs.

After successfully establishing membraneless cellular compartments and achieving an intracellular synthesis of QDs in living *E. coli* cells, we conducted a study on the synthesis process of QDs in protein droplets in vitro. External Cd^2+^ diffuses into the protein droplets and coordinates with histidine residues in the His‐tag, leading to their immobilization (Figure S15, Supporting Information). Subsequently, through Cys and SeO_3_^2−^ reduction, we successfully synthesized QDs within the protein droplets. HRTEM imaging revealed distinctive circular shadows produced by the protein droplets, which exhibited a substantial accumulation of QDs (Figure S15B, Supporting Information). Further examination using HRTEM allowed us to characterize the crystal lattice structure of the QDs, revealing individual nanocrystals with lattice planes spaced at 0.349 nm (Figure S15C, Supporting Information). Fast Fourier transform (FFT) pattern analysis showed that QDs nanostructures synthesized with HM as a template had good crystal quality (Figure S15D, Supporting Information). The diameter of the QDs was estimated to be approximately 4 nm (Figure S16E, Supporting Information). These results demonstrated the critical templating role of the HM protein in the biosynthesis of the QDs.

Although QDs exhibited bipolar distribution within the HM *E. coli*, they still maintained good fluorescence effects. No obvious aggregation was observed at the two poles of HM/IBSCS *E. coli*, indicating that the construction of HM proteins in the subcellular interior provides the basis for a stable QDs structure. The UV–Vis spectra showed the broken *E. coli* cells exhibited a clear absorption peak at 400 nm, which can be attributed to the absorption peak of CdSe*_x_* S_1‐_ *_x_* QDs (Figure S16A,B, Supporting Information), according to the reports.^[^ ^52^, ^53^, ^62^ ^]^ We compared the fluorescence emission intensities of the nanoparticles obtained from different hybrid systems. Under bright field conditions, the extracted QDs exhibited a yellowish, suggesting a certain degree of similarity in their fluorescence emission. As shown in Figure S17C (Supporting Information), when excited with a 400 nm laser, the QDs displayed a broad emission spectrum centered around 575 nm (450–700 nm range). Compared to the HydA/IBS, the HM/IBSCS demonstrated higher fluorescence emission intensity (Figure S16D,E, Supporting Information), potentially indicating that the specific environment formed within the subcellular compartment enhances the fluorescence effect. This finding further supports the pivotal role of subcellular compartments in improving the capture and utilization of photoelectrons.

Based on the above research, we characterized the biosynthetic QDs in the construction of HM *E. coli* cells. The energy band structure of QDs was characterized through a series of physical experiments. The UV visible diffuse reflection spectra (UV–vis–DRS) were recorded and converted into Tauc plots (Figure S17A, Supporting Information). The bandgap (Eg) of QDs was determined to be 2.49 eV (Figure S17B, Supporting Information). The XPS valence band was recorded and the minimum valence band (VB) was determined to be 2.07 eV (Figure S17C, Supporting Information). Even if the minimum conduction band (CB) value (−0.42 eV) was obtained, it was believed that it can catalyze various biological reduction reactions, including reduction, H^+^/H_2_ = 0 V, intracellular NAD^+^/NADH = −0.32 V (Figure S17D, Supporting Information). These results indicate that in the HM/IBSCS, QDs have good photosensitive activity and can generate sufficient photoelectrons.

### 2.3 Hydrogen Production Performance of Inorganic–Biological Subcellular Compartment Hybrid System

**Figure 5:**
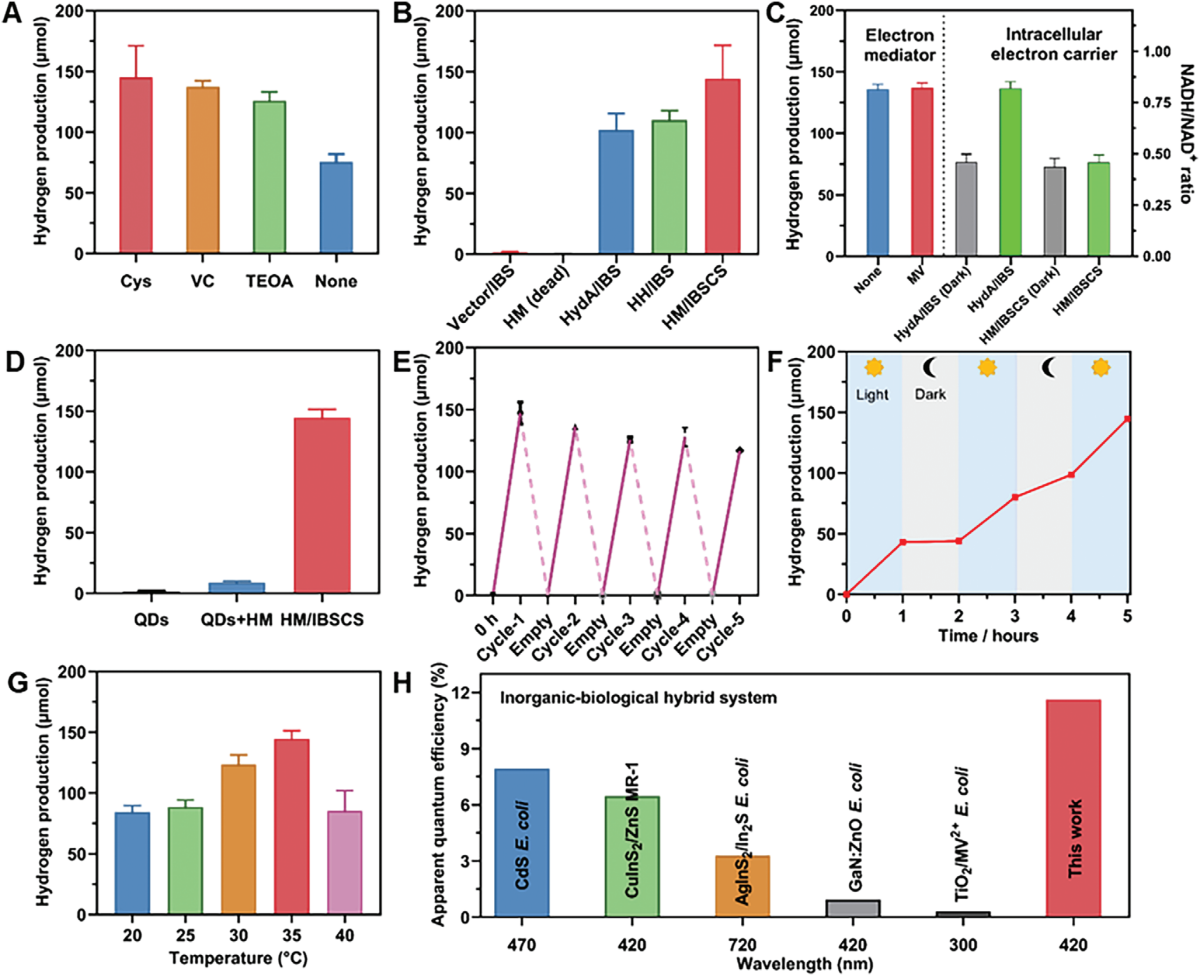
The photocatalytic hydrogen production ability of HM/IBSCS. A) Hydrogen production by HM/IBSCS in different electron donor systems under visible‐light irradiation. B) Hydrogen production of the different biohybrid under visible light irradiation. C) Hydrogen production over various electron mediators under visible light irradiation. D) Hydrogen production by the QDs, extracellular photosensitized biohybrid system (QDs+HM), and HM/IBSCS under visible light irradiation. E) The cyclic hydrogen production capacity of the HM/IBSCS. After 3 h of photocatalytic hydrogen production, nitrogen replacement is performed on the gas in the system. F) Hydrogen production by HM/IBSCS during light on/off cycles. G) Comparison of photocatalytic hydrogen production ability of HM/IBSCS at different temperatures. H) Comparison of apparent quantum yields of different biohybrid systems. Data are presented as mean ± S.D.

One of the primary objectives in constructing the IBSCS was to establish localized photosynthetic catalytic centers within living cells, aiming to enhance hydrogen gas production. We investigated the photoresponse of the hybrid system under visible irradiation and its hydrogen‐generating performance. Under visible light irradiation, the hydrogen production performance of the HM/IBSCS was evaluated. First, as shown in **Figure** **5**A, when ascorbic acid (VC), triethanolamine (TEOA), and Cys were used as sacrificial agents (SR), they effectively enhanced biologically driven hydrogen production in the hybrid system (Figure 5A). Cys, acting as a typical hole scavenger (2Cys + *2h^+^* → Cyss + 2H^+^),^[^ ^38^, ^63^ ^]^ exhibited a 192% increase in hydrogen production compared to the no sacrificial agent group when used in a mixed electron donor system. The hydrogen production from TEOA and VC increased by 168% and 182%, respectively (Figure 5A). Notably, the effect of intracellular reductants was more pronounced than TEOA. Among all experimental groups, the Cys demonstrated the best performance. This group was selected for subsequent performance evaluations.

The results indicated that the engineered *E. coli*: Biohybrid system outperforms the *E. coli* with no hydrogenase expression (Vector/IBS) in terms of hydrogen production capability (Figure 5B). Specifically, the hydrogen production capability of the HydA/IBS was 60 times than that of pure *E. coli* stain without hydrogenase expression, suggesting that introducing more hydrogenase into the bacteria through genetic engineering allowed for more efficient capture of photoelectrons from QDs, thereby effectively enhancing hydrogen production. Furthermore, the hydrogen production capability of the HM/IBSCS was 87 times higher than that of *E. coli* without hydrogenase expression (Vector/IBS). Compared to the hydrogen production of 110 µmol in the HydA/IBS after 3 h incubation, the HM/IBSCS produced 147 µmol of hydrogen, representing a 44% increase. Finally, the *E. coli* that died after heat treatment lost all its hydrogen production activity, indicating the necessity of combining inorganic semiconductors with living *E. coli* (HM dead). These results further validated the notion that the subcellular compartment constructed within living cells played a pivotal role in efficiently producing hydrogen. Additionally, an HH/IBS was further constructed to assess its hydrogen‐producing ability, which showed a slight increase in hydrogen production compared to the HydA/IBS. This indicated that the ability to form subcellular compartments and template effects were lost in the absence of MaSpI8/IDPs, but the presence of the His‐tag allowed for the binding of a small number of QDs within the *E. coli* cells.

Methylviologen (MV) is typically used in extracellular suspension/membrane‐bound photosensitive biohybrid systems to accelerate electron transfer.^[^ ^64^ ^]^ The hydrogen generation amount was almost unchanged compared to the group without the addition of an electron mediator (Figure 5C). This further confirms that the interaction between QDs and enzymes did not rely on extracellular redox mediators. The extracted bio‐QDs (whose concentration was determined based on ICP‐AES results exhibited a hydrogen production of 1.76 µmol under the same conditions, indicating a synergistic effect between bio‐QDs and bacteria (Figure 5D). When comparing the extracted QDs (QDs + HM, 8.96 µmol) physically mixed with *E. coli*, the hydrogen production only increased by 5.07 times. However, in the case of the HM/IBSCS, the hydrogen production increased by a remarkable 83.46 times. These results demonstrated that the precise binding of the HM protein within the *E. coli* cell to the QDs significantly enhances the hydrogen production capacity of the system. We further investigated the sustained hydrogen production capability of the HM/IBSCS. After 5 cycles of continuous photo‐catalytic hydrogen production over 15 hours, the total hydrogen production reached 650.51 µmol (Figure 5E). Even after the fifth cycle, approximately 81% of the hydrogen production capacity was retained, highlighting the system's robustness and longevity in hydrogen generation. Further light‐dark alternation experiments have verified the importance of light in HM/IBSCS (Figure 5F). LLPS generally exhibits temperature dependence, with elevated temperatures potentially stabilizing or destabilizing the LLPS of intrinsically disordered proteins.^[^ ^65^ ^]^ However, in our study, we observed that within the temperature range of 20–40 °C, the compartments within bacterial cells did not undergo significant changes (data not shown). Furthermore, we further assessed the hydrogen production capacity of *E. coli* expressing hydrogenase in a glucose‐supplemented buffer (Figure S18, Supporting Information), revealing temperature‐dependent hydrogen generation. Subsequent experiments demonstrated that the HM/IBSCS hydrogen production capability remained consistent with the trend observed for biological fermentation hydrogen production at different temperatures (Figure 5G). These results collectively indicated that compartments constructed using MaSpI8/IDPs as living cell scaffolds were the least sensitive to temperature fluctuations within this range. This may be because the phase transition of spider silk protein is less affected by temperature.^[^ ^66^ ^]^ In addition, the effect of temperature on hydrogen production in HM/IBSCS was consistent with the trend observed in traditional fermentation hydrogen production methods. This consistency was attributed to the fact that temperature had a greater impact on enzyme activity than on compartment stability. Finally, we evaluated and compared the photocatalytic hydrogen production performance in typical biological hybrid systems using different methodologies (Figure 5H and Tables S3 and S4, Supporting Information). Compared with energy conversion efficiency, IBSCS exhibits higher apparent quantum efficiency (AQE) in inorganic biological hybrid systems. Compared with nonhybrid systems/photosensitizers, IBSCS has the highest reported hydrogen production performance.

### 2.4 Hydrogen Production Mechanisms and Biocompatibility of Hybrid System

**Figure 6:**
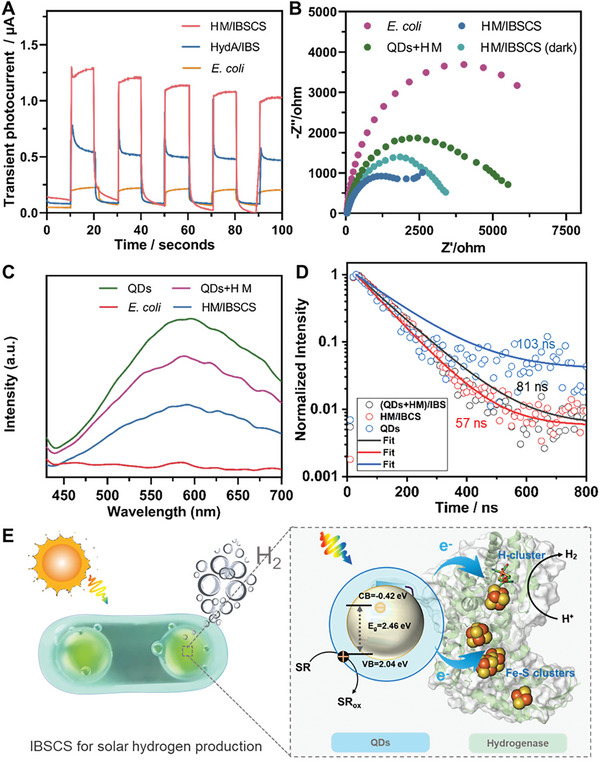
Electron transfer mechanism in HM/IBSCS. A) Transient photocurrent responses of *E. coli*, HydA/IBS, HM/IBSCS. B) Fitted electrochemical impedance spectra of the different biohybrid systems. C) Steady‐state fluorescence spectra of QDs, *E. coli*, QDs+HM/IBS, and HM/IBSCS (*λ*_ex_ = 400 nm). D) Time‐resolved fluorescence spectra of QDs, QDs+HM/IBS, and HM/IBSCS, fit with biexponential decay functions (solid lines). E) Structure diagram and possible electron transfer pathways. The electrons excited by QDs are transferred through a tightly integrated electron transport chain (Fe–S clusters) to a catalytic center (H‐clusters), ultimately catalyzing the production of H_2_. The enzyme structure used in the cartoon is *Clostridium pasteurianum* [Fe–Fe] hydrogenase (HydA), PDB: 3C8Y, created using PyMOL.

To gain deeper insights into the generation and transfer mechanisms of photo‐induced charge carriers for photocatalytic hydrogen production within the HM/IBSCS system, we conducted photoelectrochemical studies and steady‐state fluorescence spectroscopy measurements (**Figure** **6**). Different hybrid bacteria were immobilized on FTO electrodes, resulting in significant photocurrent generation during photo‐switching. *E. coli*, HydA/IBS, and HM/IBSCS all exhibited photocurrent under simulated solar illumination. Notably, the photocurrent was significantly enhanced in the presence of subcellular mineralized QDs within HM/IBSCS, surpassing that of the HydA/IBS and *E. coli* alone (Figure 6A). The finding underscored the efficient light‐capturing ability conferred by the subcellular mineralized QDs.^[^ ^67^ ^]^ The better photoelectric performance of QDs can mean that a higher flux of electrons is available for hydrogenase, thereby enhancing the efficiency of hydrogen production within biological organisms. Metal nanoparticles have been explored to reduce charge transfer resistance and enhance charge transfer efficiency.^[^ ^68^ ^]^ We conducted electrochemical impedance spectroscopy (EIS) to investigate the role of QDs in the charge transfer process.^[^ ^69^ ^]^ Notably, according to the semicircle size in Figure 6B, the charge transfer resistance of HM/IBSCS is significantly lower than that of the hybrid system involving *E. coli* and extracellular photosensitizer (QDs+HM). Under illumination conditions, the charge transfer resistance of HM/IBSCS further decreased, which confirmed that the photoexcited charge separation of QDs enhances electron flow between *E. coli* and electrodes. This improvement could be attributed to the enhanced quantity and quality of QDs,^[^ ^70^ ^]^ especially those mineralized with MaSpI8/IDPs as templates, exhibiting superior photoelectrochemical performance. The reduced charge transfer resistance facilitated efficient electron transfer, and the subcellular compartments formed by HM and QDs condensates contributed to the construction of an efficient electron transport network. Besides, electron transfer in HM/IBSCS using steady‐state fluorescence spectroscopy indicated no significant fluorescence changes were observed in pure *E. coli* cells. In contrast, the photoluminescence (PL) intensity slightly decreased in the extracellularly added QDs group ((QDs+HM)/IBS), while a significant reduction in PL intensity was observed in HM/IBSCS. This indicated that electrons generated by QDs in HM/IBSCS were transferred to the electron acceptor hydrogenase, suppressing the recombination of electrons with holes and reducing outward photoluminescence (Figure 6C). Time‐resolved fluorescence spectra of QDs, (QDs+HM)/IBS and HM/IBSCS showed that the fluorescence lifetime of 57 ns was shorter than other groups, indicating that the photo‐induced electrons were transferred to hydrogenases. Previous research findings have demonstrated that the introduction of the intracellular electron mediator (MV^2+^) did not alter hydrogen production in the existing system (Figure 5C), this indicates that the production of hydrogen may occur through direct electron transfer between QDs and hydrogenases. In the extracellular and cytoplasmic photosensitive hybrid systems, the NADH/NAD^+^ levels represent the directly generated reduction equivalents from photocatalytic reactions.^[^ ^54^, ^63^ ^]^ Subsequently, enzymes further utilize these reduction equivalents to generate reaction products, rather than directly transferring the photoexcited electrons to enzymes.^[^ ^71^, ^72^ ^]^ This increases the uncertainty in electron transfer, leading to poor specificity in product formation, and may even result in an upregulation of metabolites throughout the entire NADH metabolic pathway, resulting in a low energy utilization efficiency. We further examined the NADH/NAD^+^ levels in different hybrid systems under both light and dark conditions. We observed a significant increase in the content of HydA/IBS under light conditions, while HM/IBSCS showed only a slight increase (Figure 5C). Despite HM/IBSCS generating a higher number of photoelectrons (Figure 6A), there was only a limited reduction of NAD^+^. These findings confirm that in the IBSCS system, electrons produced by the light‐excited QDs are directly transferred to hydrogenase.^[^ ^15^ ^]^ Under natural conditions, the Fe‐S clusters attached to the hydrogenase act as mediators, transferring high‐energy electrons to the H‐cluster, ultimately reducing H^+^ to H_2._^[^ ^73^, ^74^ ^]^ Within the subcellular compartments formed by the HM protein, 2×His tags are located at the C‐terminus of the hydrogenase subunit and are located in the middle of the Fe‐S electron transfer chain.^[^ ^75^, ^76^ ^]^ Due to the proximity between the His‐tags and the H‐cluster/Fe–S clusters, direct electron injection from photo‐activated QDs is anticipated to occur (Figure 6E). This comprehensive study provides valuable insights into the mechanisms of photo‐induced charge carrier generation and transfer within the HM/IBSCS system, elucidating its potential for efficient hydrogen production through semi‐artificial photosynthesis.

In the activity test of bacteria, the colony forming unit (CFU) was measured to evaluate the activity of bacteria after photocatalytic reaction, and the results confirmed the superior biocompatibility of biosynthetic QDs (Figure S19A,B, Supporting Information). We employed a fluorescent probe specific to reactive oxygen species (ROS) to measure fluorescence emission intensity, allowing us to assess the ROS levels within the HM/IBSCS. As shown in Figure S19C,D (Supporting Information), the fluorescence intensity remained relatively consistent before and after light exposure, indicating comparable ROS levels under both conditions, proving that the presence of the HM/IBSCS did not significantly alter the intracellular ROS balance, thus avoiding disruption to the normal metabolic functions of the bacteria. As holes are effectively quenched by sacrificial agents, photoelectrons are directed toward hydrogenase to catalyze hydrogen gas production.^[^ ^72^, ^77^ ^]^ It is worth noting that despite the potential toxicity of metal nanoparticles and ROS generated through light‐induced processes, in our constructed HM/IBSCS, these adverse factors have been minimized for several reasons. On one hand, the bio‐QDs are coated with a naturally formed protein layer composed of glutathione and cysteine,^[^ ^78^ ^]^ which imparts excellent biocompatibility to the QDs and provides substantial ROS quenching capacity. On the other hand, the compartment confined the photocatalytic reaction within a specific region, thus preventing the diffusion of photo‐generated holes. This localized reaction effectively mitigated the potential adverse effects that may arise from species generated by photoexcitation.

## 3 Conclusion

Liquid–liquid phase separation (LLPS) serves as a versatile tool widely employed in cellular processes, regulating diverse biological phenomena through the dynamic formation of liquid droplets. In summary, this study establishes a proof‐of‐concept and demonstrates an innovative approach utilizing LLPS to create a membraneless subcellular organelle for intracellular semi‐artificial photocatalysis. Recombinant hydrogenase, combined with biocompatible QDs produced through cellular biomineralization, forms subcellular compartments for semi‐artificial photocatalysis. In the catalytic process, electrons generated by intracellular quantum dots directly transfer to hydrogenase, achieving efficient hydrogen production and avoiding electron losses during transmembrane transport. The subcellular compartments effectively confine the migration of high‐energy electrons and holes, preventing the generation of reactive oxygen species (ROS) and enhancing system stability. Notably, *E. coli*, as one of the most common bacteria, provides a robust foundation for the generality of our approach. Therefore, this work thus represents an updated exploration of a semi‐artificial photosynthetic system with advanced synthetic biology and sustained hydrogen production. This system holds promise for extension into research areas such as carbon fixation, nitrogen fixation, and cascading enzyme reactions, providing more precise, efficient, and controllable methods for the fields of biosynthesis and energy conversion, offering new insights for future research and applications.

## 4 Experimental Section

### Amino acid Sequence Analysis

Prediction of intrinsically unstructured proteins was performed using the IUPRed2 program (https://iupred2a.elte.hu/).

### Strain Construction

All plasmids and protein sequences used in this study are listed in Tables S1 and S2 (Supporting Information), which were constructed according to previously reported methods.^[^ ^14^, ^33^, ^34^ ^]^ A dual vector expression system was used in which pETDuet‐1 and pCDFDuet‐1 contained the HydA protein fused to silk protein (MaSpI8/IDPs) and its maturase HydE, HydF, and HydG (named as Strain1/HM). HydE, HydF, and HydG play an irreplaceable role in the H‐cluster biosynthesis of hydrogenase (HydA) as a group of Fe‐S “maturase” enzymes.^[^ ^79^ ^]^ Similarly, the HydA‐2×His‐tag group was handled in the same way (Strain3/HH). The construction of the active hydrogenase (HydA) was achieved by transforming pETDuet‐1 and pCDFDuet‐1 with HydAE and HydFG, respectively, and then transferring them into *E. coli* BL21 (DE3) as a control (Strain2/HydA). Two empty vectors were transfected into *E. coli* BL21 (DE3) as negative controls (Strain4/Vector). For a more visual and intuitive design, refer to Support Information.

### HydA‐MaSpI8 Fusion Protein (HM) Purification, Quantification, and SDS‐PAGE Analysis

Cell growth was monitored by measuring the absorbance at 600 nm (OD600). At an OD600 of ≈0.6, the culture temperature was shifted to 20 °C, and the bacterial cells were treated with 0.1 × 10^−3^ m IPTG to induce hydrogenase and silk protein production for 24 h. Then, the cells were analyzed on 15% SDS‐PAGE gels and were harvested for protein purification. *E. coli* in lysis buffer (20 mM Tris‐HCl, pH 7.4, 100 × 10^−3^ m NaCl, and 10 × 10^−3^ m imidazole) was lysed by sonication. After centrifugation, the supernatant was loaded onto a Ni‐NTA column. The column was washed with 5 mL washing buffer twice (20 × 10^−3^ m Tris‐HCl, pH 7.4, 100 × 10^−3^ m NaCl, and 50 × 10^−3^ m imidazole) and then eluted with elution buffer (20 × 10^−3^ m Tris‐HCl, pH 7.4, 100 × 10^−3^ m NaCl, and 250 × 10^−3^ m imidazole). In addition, the purified samples of His‐tag were further desalinated and concentrated using 50K ultrafiltration tubes. The protein concentrations were determined by using the BCA Protein Assay kit (Sangon Biotech).

### Dextran‐70‐Induced Phase Separation In Vitro

At room temperature, quickly invert and mix 50 mg mL^−1^ of dextran 70 macromolecule crowding agent with 50 × 10^−6^ m of HM/HH protein. Subsequently, observe the generation of protein droplets under a microscope. The centrifugal rotation displayed separates into two liquid phases, and the precipitation/condensation phase was characterized by SDS‐PAGE.

### Biomineralization of QDs in *E. coli*

Modified based on the previously demonstrated strategy for bacterial intracellular biosynthesis of CdSe*_x_* S_1‐_ *_x_* nanoparticles.^[^ ^33^, ^52^, ^54^ ^]^ After the hydrogenase and silk protein production for IPTG‐induced for 24 h, the cells were then exposed to 1 × 10^−3^ m CdCl_2_ and 1 × 10^−3^ m Na_2_SeO_3_ in anaerobic conditions, and cultured for an additional 3 h for the biosynthesis of QDs. The color of the *E. coli* cell precipitation changed from white to yellow, indicating the formation of the biohybrid. Finally, the bacterial culture was harvested by centrifugation at 10 000 rpm for 1 min and resuspended in 20 mL of Tris buffer with an additional 50 × 10^−3^ m glucose supplemented as an energy source. Cadmium concentration was determined by inductively coupled plasma‐atomic emission mass spectrometry (ICP‐5000, China).

### Confocal Imaging of *E. coli* Cells

Confocal imaging was performed on a Zeiss LSM 900 (Zeiss, Germany). The *E. coli* cells synthesizing bio‐QDs were collected by centrifugation (5000 rpm, 4 °C, 5 min), and washed with PBS buffer twice. The bacterial cells were then resuspended with fresh PBS and transferred (≈5 µL) onto glass slides. The specimens were illuminated with a diode 488 nm laser for QDs fluorescence imaging. In another setup, the HM protein/*E. coil* was stained with 100 µg mL^−1^ ThT, a fluorescent dye that probes the β‐sheet structures.

### Transmission Electron Microscope (TEM) Measurement

The bacteria mineralized by QDs (OD600 ≈0.8) were centrifuged and washed three times with Tris–HCl buffer. After confocal verification, they were fixed overnight with a 4% paraformaldehyde solution at 4 °C and dehydrated for 20 min using a gradient concentration ethanol aqueous solution (20%, 40%, 60%, 80%, 90%, and 100%).^[^ ^80^ ^]^ The sample was dropped onto a 200‐mesh copper mesh for TEM characterization (FEI Talos F200S). The morphologies of thin‐sectioned *E. coli* were characterized by Bio‐transmission electron microscopy (Bio‐TEM, Hitachi HT7800, 80 kV). The *E. coli* sample was further baked at 70 °C for 12 h for complete curing and cut into 70 nm thin sections with a diamond knife.

### Dynamic Light Scattering

50 × 10^−6^ m protein solution and 50 mg mL^−1^ dextran‐70 solution were centrifuged at high speed and mixed well. Analyze 1 mL of mixed solution using a Zetasizer Nano DLS instrument (Malvern Instruments). Measure 10 rounds for 10 s each time, and use Zetasizer software for data processing.

### Fluorescence Recovery After Photobleaching

Fluorescence recovery after photobleaching experiments was conducted with a Zeiss LSM 900 (Zeiss, Germany). The HM protein was set as 50 × 10^−6^ m and ThT was 100 µg mL^−1^. The ThT signal was bleached using a 488 nm laser beam. The fluorescence intensity within the selected region was monitored and normalized to the intensity before bleaching.

### Transient Photocurrent and Electrochemical Impedance Spectroscopy (EIS) Measurement

Centrifuge to collect 50 mg hybrid bacterial samples from different systems. Subsequently, mix the precipitated bacterial cells with 100 µL of Tris buffer. Uniformly apply the obtained mixed bacterial suspension onto the surface of an FTO glass electrode, then air‐dry to prepare a working electrode. A Pt wire and an Ag/AgCl electrode were used as the counter and reference electrodes, respectively.^[^ ^81^, ^82^ ^]^ Tris‐HCl buffer was used as the electrolyte. The solution was purged by N_2_ flow for 30 min. The photocurrent was recorded using a CHI‐660E electrochemical workstation (CHI650E, CH Instruments, USA), with 350–780 nm visible light (100 mW cm^−2^) used as the external light source. EIS measurement was carried out at an open‐circuit potential without external bias, covering a frequency range from 10^5^ to 0.1 Hz.

### Reconstituted Biosynthesis of the QDs In Vitro

Biosynthesis of the QDs was performed by the previous protocol.^[^ ^33^ ^]^ Using the purified HM as a template in 50 × 10^−3^ m Tris‐HCl buffer. After thoroughly mixing 50 × 10^−6^ m CdCl_2_ with HM and incubating at 25 °C for 30 min, an additional 40 × 10^−6^ m Na_2_SeO_3_ is introduced, followed by a further 30 min incubation to facilitate the formation of QDs. Characterize the QDs image of the reaction mixture using TEM (FEI Talos F200S).

### Evaluation of Hydrogen Production Performance of the Hybrid

To evaluate the photocatalytic hydrogen production performance of the inorganic‐biohybrid, a certain amount of hybrid cell was added into a glass tube containing 20 mL biohybrid solution (100 × 10^−3^ m Tris‐HCl buffer pH = 7, 2 × 10^−3^ m cysteine (Cys) as the sacrificial agent (10 × 10^−3^ m ascorbic acid (VC), 10 × 10^−3^ m triethanolamine (TEOA)) and 50 × 10^−3^ m glucose (as the carbon source). In a specific experiment, the concentration of the electron mediator MV^2+^ is 5 × 10^−3^ m. The accumulative hydrogen content in the gaseous phase was detected regularly using a gas chromatograph (Agilent GC‐8890, USA). All assays were performed anaerobically on 42 mL serum glass bottles with a working volume of 20 mL and nitrogen as gas headspace. The photocatalytic experiments were carried out using a 300 W Xenon lamp (Aulight, Beijing) that emits an irradiance of 100 mW cm^−2^ (350–780 nm). The reaction temperature was controlled using a circulating water bath. In addition, the apparent quantum efficiency of the hybrid, which describes the proportion of photoelectrons being utilized for hydrogen production, was measured under the incident light of 420 nm wavelength and 10 mW cm^−2^ intensity. The AQE was calculated based on a previously described approach.^[^ ^51^ ^]^ At the fixed time point of the hydrogen production experiments, a 1 mL gaseous sample from the headspace of the reactor was collected and measured by gas chromatography. The hydrogen concentration was quantified by putting the measured peak area into the standard curve. Finally, the original hydrogen concentration unit was transformed into µmol. In the hydrogen generation process, low‐speed magnetic stirring was applied to inhibit the settling of the sample. All samples were prepared in an anaerobic glove box.

### Cell Viability, ROS, and NADH/NAD^+^ Ratio Measurement

The cell viability was determined by MTT 3‐(4,5‐dimethylthiazol‐2‐yl)−2,5‐diphenyltetrazolium bromide tetrazolium assay.^[^ ^83^ ^]^ After the irradiation experiment, the viability of *E. coli* was determined by colony‐forming units’ assays (CFU). 100 µL of the collected sample was serially diluted with saline. 100 µL of the original and diluted samples were spread on sterilized nutrient agar plates and then incubated at 37 °C for 16 h. Finally, the survival number of cells was quantified by counting the visible colonies, and the survival rate was calculated. The intracellular level of ROS was measured using a fluorescent dye molecular probe 2′,7′‐dichlorofluorescein diacetate (DCFH‐DA, Sangon Biotech).^[^ ^78^, ^84^ ^]^ Cultures were incubated with 100 µM DCFH‐DA at 37 °C for 30 min. After incubation, *E. coli* cells were collected by centrifugation. A fluorescence excitation wavelength of 488 nm was used to detect the ROS produced in the sample. The determination of the NADH/NAD^+^ ratio within bacteria is based on the manufacturer's instructions (China, NJJCBIO, A114‐1‐1).

### Statistical Analysis

Data statistical analysis was conducted using GraphPad 8 software (USA). The data presentation was shown as the mean ± SD.

## Conflict of Interest

The authors declare no conflict of interest.

## Supporting information

Supporting Information

## Data Availability

The data that support the findings of this study are available in the Supporting Information of this article.
