# Supplementary material for: Liquid–Liquid Phase Separation‐Mediated Photocatalytic Subcellular Hybrid System for Highly Efficient Hydrogen Production
Source: Adv Sci (Weinh). 2024 Apr 4;11(22):2400097. doi: 10.1002/advs.202400097 (PMC11165473; doi:10.1002/advs.202400097)
Supplement: Supplementary file 1 — Supporting Information [file ADVS-11-2400097-s001.pdf]

ADVANCED SCIENCE
Open Access

Supporting Information

for *Adv. Sci.*, DOI 10.1002/advs.202400097

Liquid–Liquid Phase Separation-Mediated Photocatalytic Subcellular Hybrid System for Highly Efficient Hydrogen Production

Xiaoxuan Yu, Hui Li, Chengchen Xu, Zhengwei Xu, Shuheng Chen, Wang Liu, Tianlong Zhang, Hongcheng Sun, Yan Ge*, Zhenhui Qi* and Junqiu Liu**

Supporting Information for

Liquid-Liquid Phase Separation-mediated Photocatalytic Subcellular Hybrid System for Highly Efficient Hydrogen Production

Xiaoxuan Yu[1,2], Hui Li[1,2], Chengchen Xu[1], Zhengwei Xu[1], Shuheng Chen[1], Wang Liu[1], Tianlong Zhang[1], Hongcheng Sun[1,], Yan Ge[2,*], Zhenhui Qi[1,2,*], Junqiu Liu[1,*]*

1 Key Laboratory of Organosilicon Chemistry and Material Technology, Ministry of Education, College of Material, Chemistry and Chemical Engineering, Hangzhou Normal University, Hangzhou 311121, China

*E-mail: sunhc@hznu.edu.cn; junqiuliu@hznu.edu.cn

2 Sino-German Joint Research Lab for Space Biomaterials and Translational Technology, School of Life Sciences, Northwestern Polytechnical University, Xi'an 710072, China

*E-mail: ge@nwpu.edu.cn; qi@nwpu.edu.cn;

This PDF file includes:

Figures S1 to S19

Tables S1 to S4

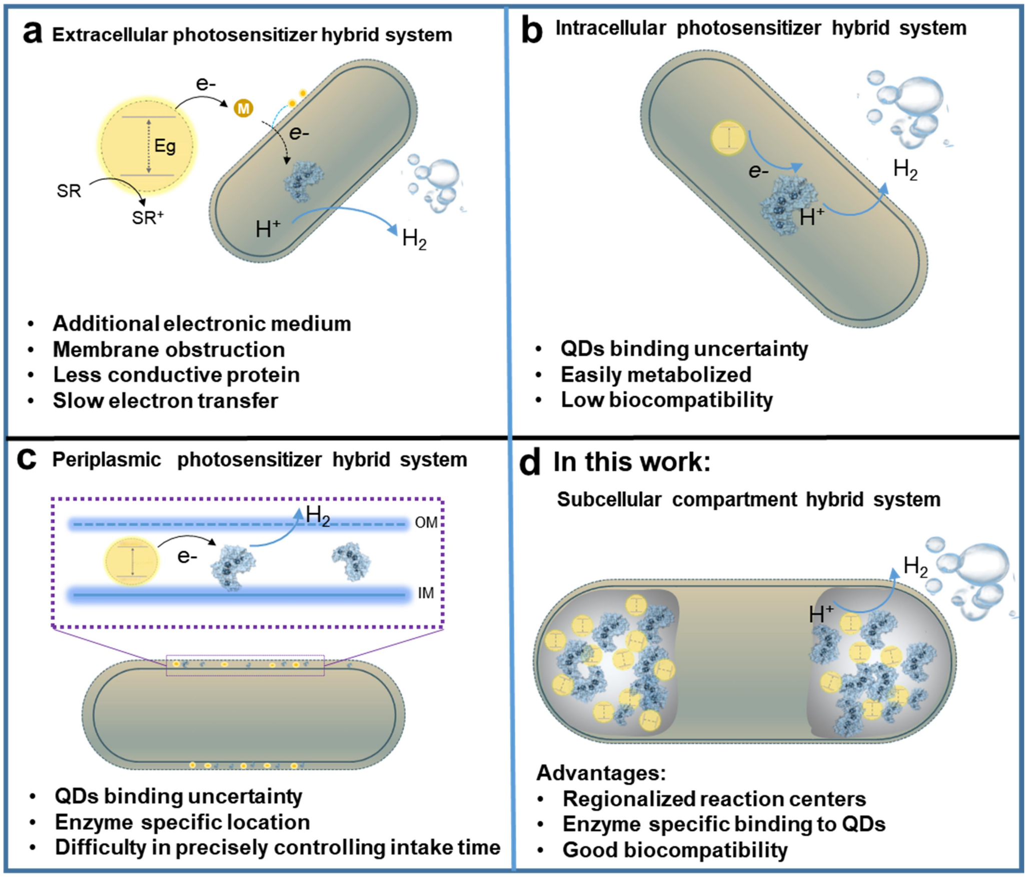

Fig S1. Typical inorganic biological hybrid systems (IBS) for solar hydrogen production. (a) Extracellular photosensitizer hybrid system. (b) Intracellular photosensitizer hybrid system. (c) Periplasmic photosensitizer hybrid system. (d) Inorganic biological subcellular compartment hybrid system (IBSCS) proposed in this work.

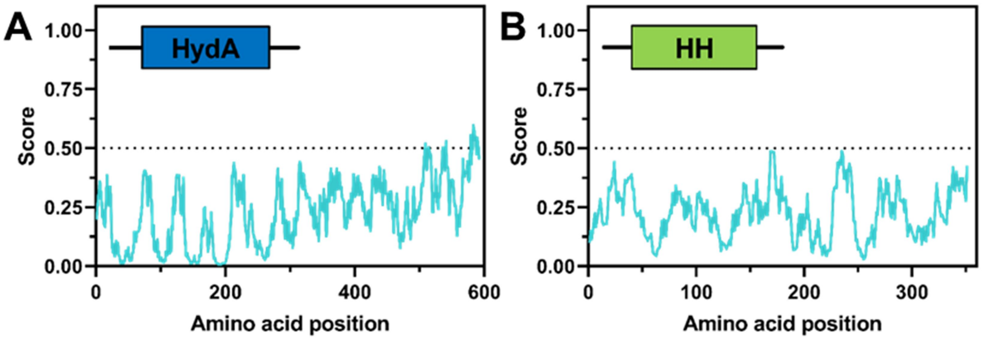

Figure S2. Prediction of protein disorder of (A) hydrogenase (HydA), (B) hydrogenase with his-tag (HH) using the IUPRed2 program. A score greater than 0.5 (above the horizontal line) indicates an unordered region, while a score less than 0.5 indicates an ordered region. The fusion of hydrogenase with MaSpI8 IDPs theoretically possesses the capability for LLPS.

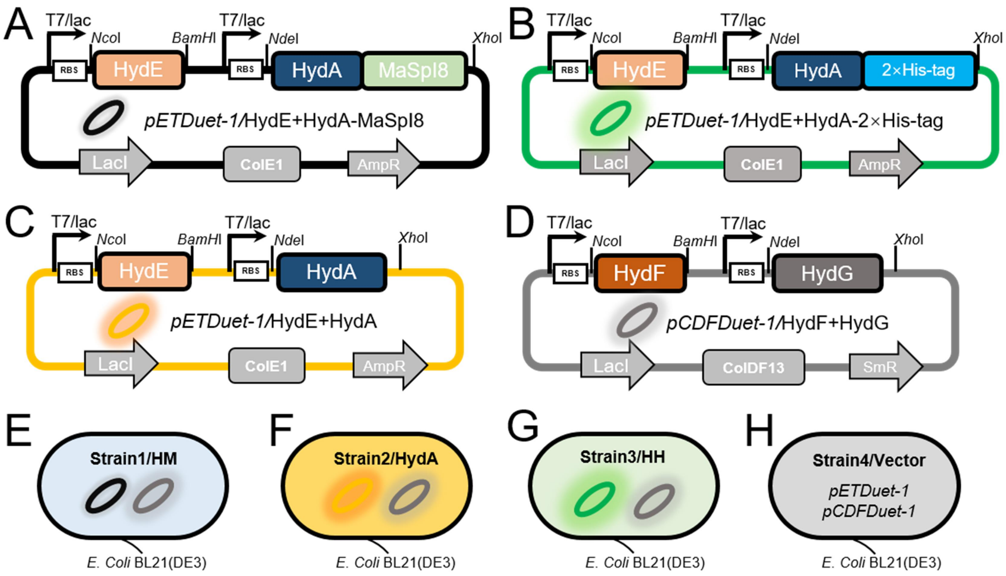

Figure S3. An optimized recombinant hydrogenase expression system was constructed based on a dual-plasmid system and *E. coli* BL21(DE3). (A) The gene construct diagram illustrates the fusion expression of the silk protein (MaSpI8) at the C-terminus of HydA (HM). (B) A schematic diagram demonstrates the fusion expression of a fragment containing two His-tags with the C-terminus of HydA. (C) HydE and HydA are separately cloned into two multiple cloning sites (MCS) of the *pETDuet-1* vector, (D) while HydF and HydG are cloned into another dual MCS of the *pCDFDuet-1* vector.

The presence of HydEFG is essential for the production of active hydrogenase. (E) **Fusion enzymes: Strain1/HM** (*pETDuet-1*/HydE+HydA-MaSpI8 + *pCDFDuet-1*/HydF+HydG). (F) **Free enzymes (as control): Strain2/HydA** (*pETDuet-1*/HydE+HydA + *pCDFDuet-1*/HydF+HydG), (G) **Strain3/HH:** *pETDuet-1*/HydE+HydA-2×His-tag + *pCDFDuet-1*/HydF+HydG). (H) **Strain4/Vector:** The dual-empty plasmid vector is transformed into *E. coli* BL21 to construct the empty control group. RBS: ribosome-binding site.

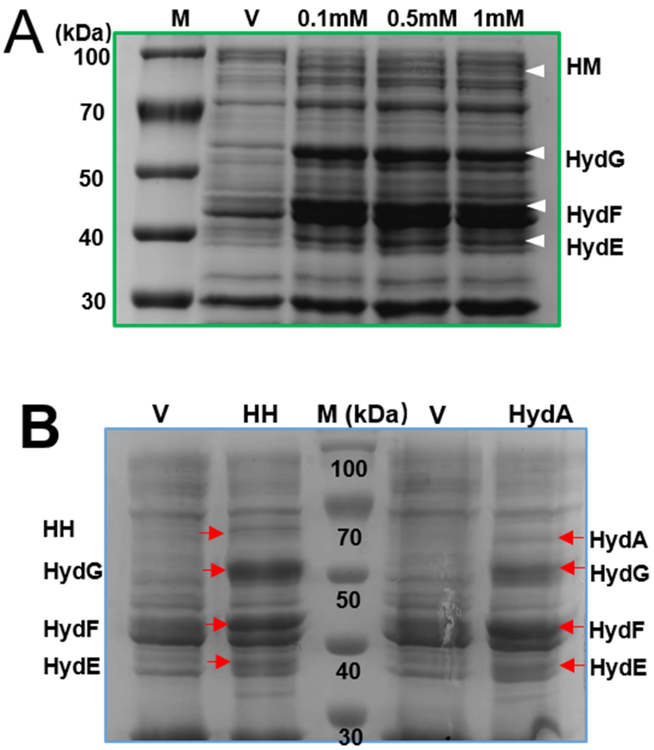

Figure S4. (A) SDS-PAGE analysis of HM and its relevant maturases (HydEFG) under varying IPTG concentrations. The concentration of IPTG is 0.1, 0.5, and 1 mM. Under 0.1 mM IPTG conditions, the expression level of hydrogenase is highest.

(B) Analysis by SDS-PAGE is performed on recombinant *E. coli* after the anoxic co-expression of HydA or HH and the HydEFG maturases. Lane M: molecular weight marker; Lane V: *E. coli* BL21(DE3) transformed with empty vector. HM has a theoretical molecular weight of 93.2 kDa, while the theoretical molecular weights of HH, HydA, HydE, HydF, and HydG are 69.1 kDa, 65.5 kDa 40 kDa, 45.8 kDa, and 53.6 kDa, respectively.

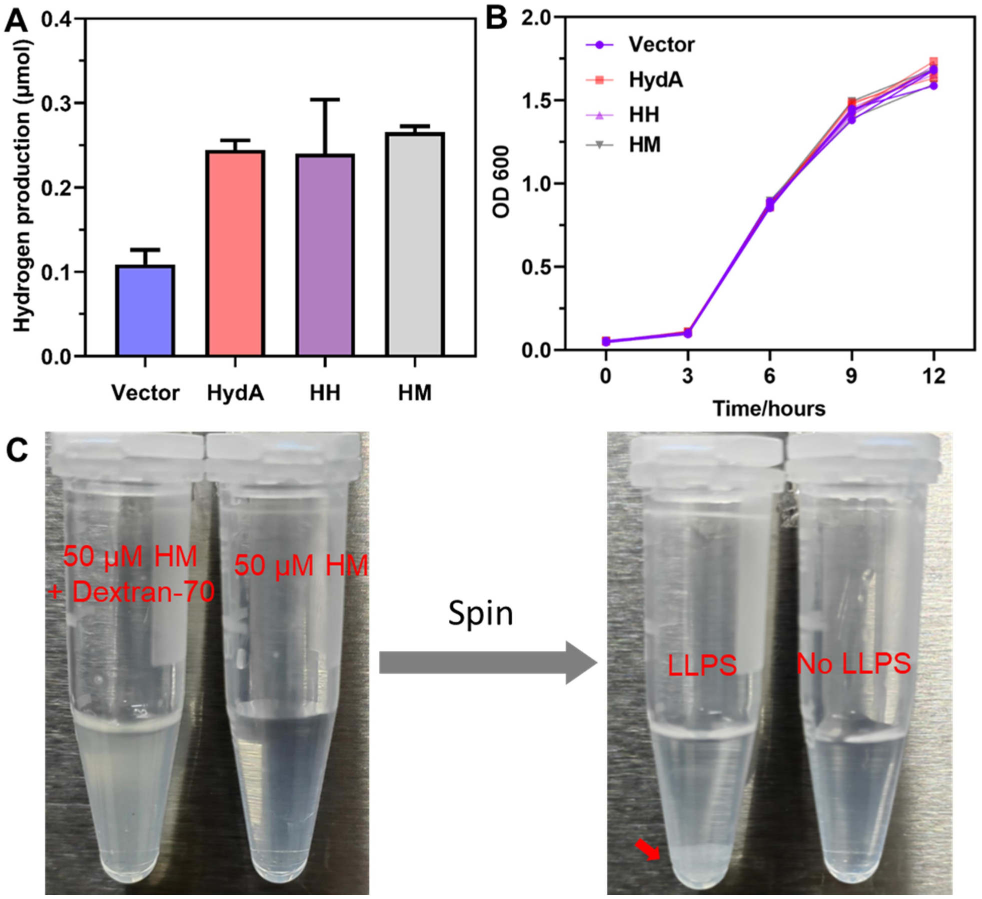

Figure S5. (A) Hydrogen production is measured by detecting the hydrogen content in sealed bottle headspace after 24 hours of induction. The results reveal that the fusion of different fragments (2×His-tag and MaSpI8) at the C-terminus of hydrogenase do not affect its hydrogen production activity. Only a small amount of hydrogen gas is produced in the growth medium. (B) Comparing the cell growth of engineered strains that have undergone genetic modifications through monitoring optical density (OD600). Similar rates of cell growth indicate that the expressed protein has no adverse impact on cell growth. (C) At room temperature, HM protein appears cloudy when mixed with the macromolecular crowding agent Dextran-70. Rotate the mixture through centrifugation, and the bottom of the centrifuge tube shows the coexistence of two liquid phases, as shown by the red arrow in the photograph.

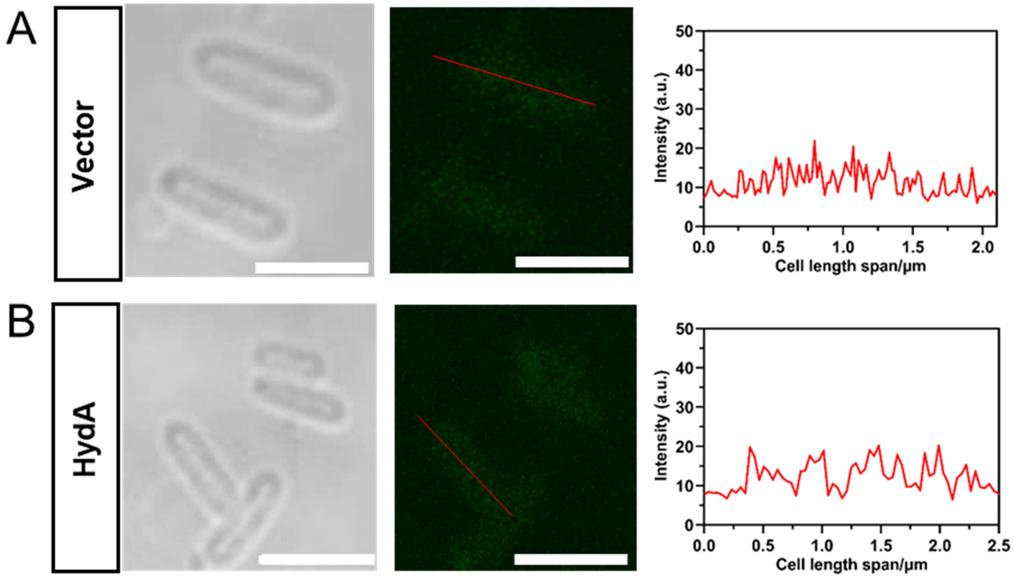

Figure S6. The bright-field and confocal laser scanning microscopy images of the *E. coli* expressing empty Vector (A) and HydA (B) stained with ThT reveal minimal green fluorescence. The fluorescence intensity line-cuts analysis showed no significant change in bacterial fluorescence.

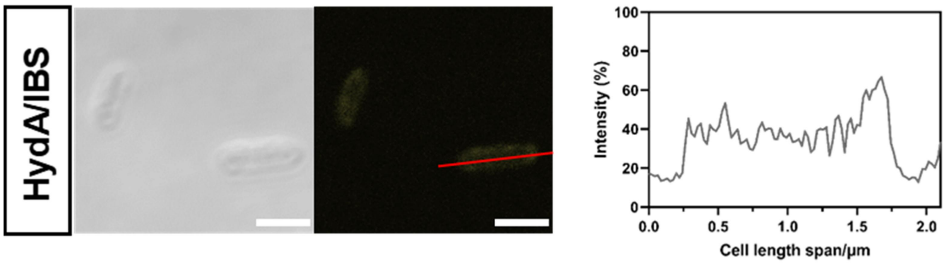

Figure S7. CLSM images for the $CdSe_xS_{1-x}$ QDs mineralized in HydA/IBS. In this biohybrid systems, mineralization of $CdSe_xS_{1-x}$ QDs occurred simultaneously and exhibited consistent fluorescence trends within the cells. Scale bar = 1 μm.

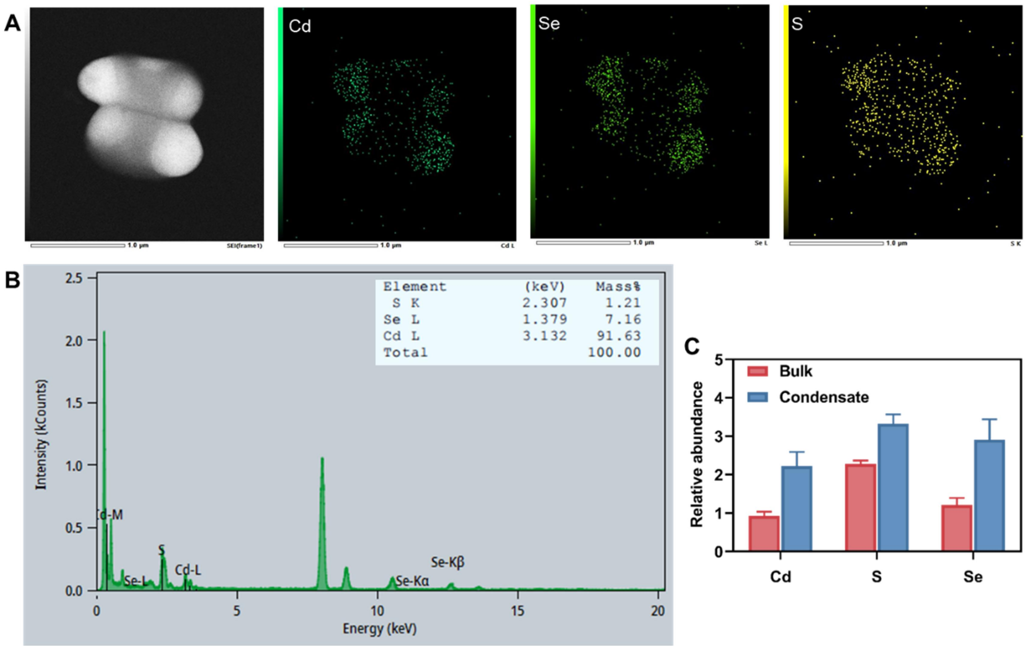

Figure S8. (A) TEM images for the $CdSe_xS_{1-x}$ mineralized HM/IBSCS. Cd, Se, and S elements exhibit spatially oriented distribution, with aggregated distribution at the two poles of *E. coli.* (B) The result of EDS analysis of the precipitated $CdSe_xS_{1-x}$ nanoparticles. (C) Relative abundance of Cd, Se, and S elements within and outside the condensates in the HM-expressing cells.

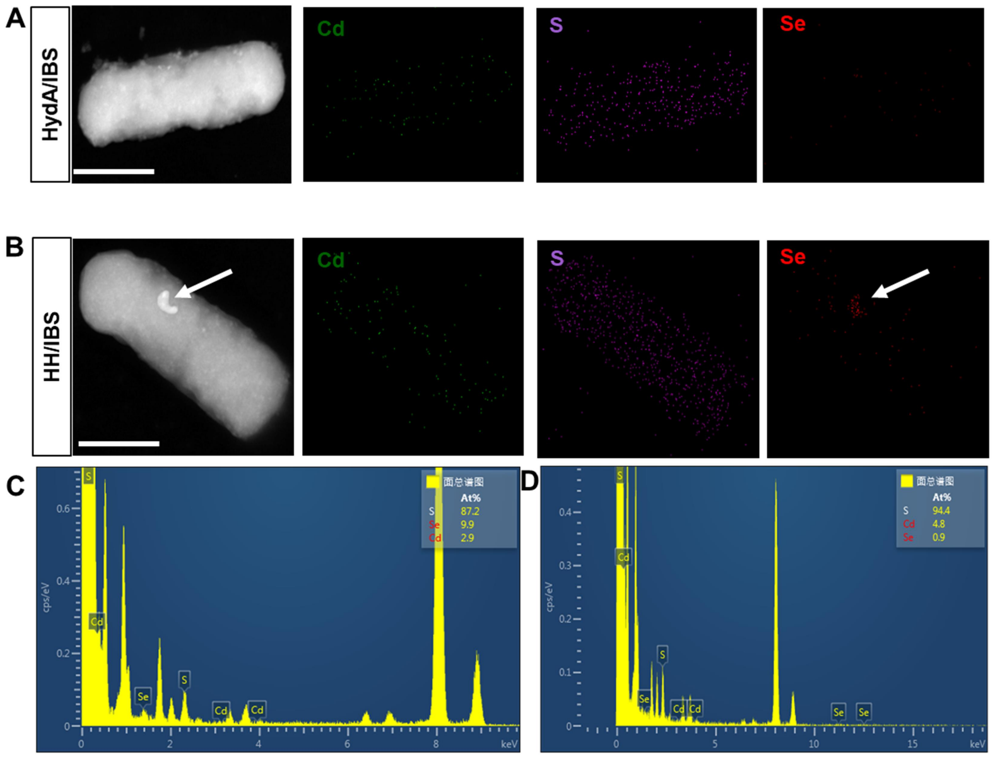

Figure S9. HRTEM images for the QDs mineralized in HydA/IBS (A) and HH/IBS (B). The result of EDS analysis of the precipitated Se particle. The white arrows pointed to the large-sized Se precipitate.

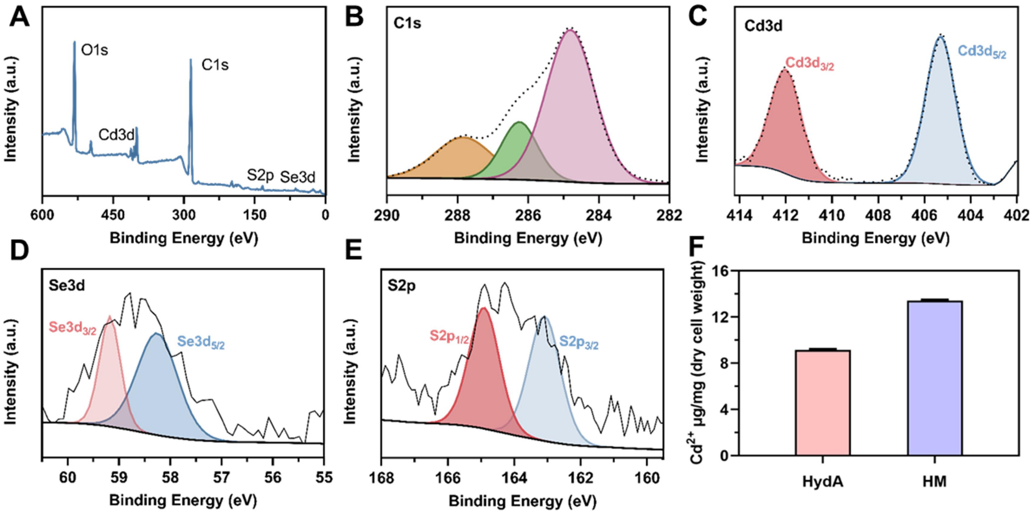

Figure S10. High-resolution XPS spectra of QDs with full spectrum (A) and fine spectra of C1s (B), Cd3d (C), Se3d (D), and S2p (E). (F) The Cd elemental concentration of the biohybrid system.

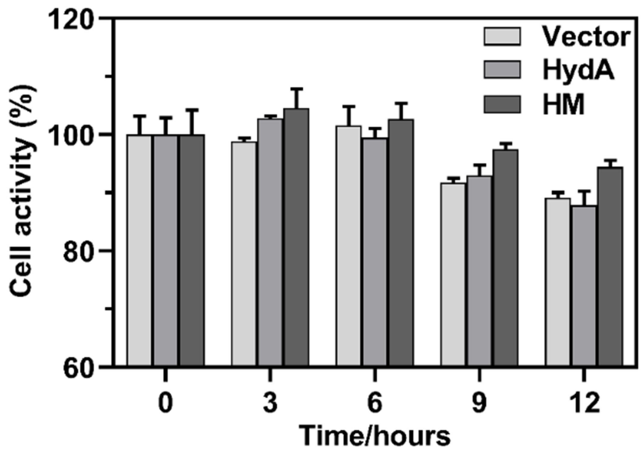

Figure S11. The MTT reduction activity was used to represent cell activity after treatment with 1 mM $CdCl_2$ and Na_2SeO_3,

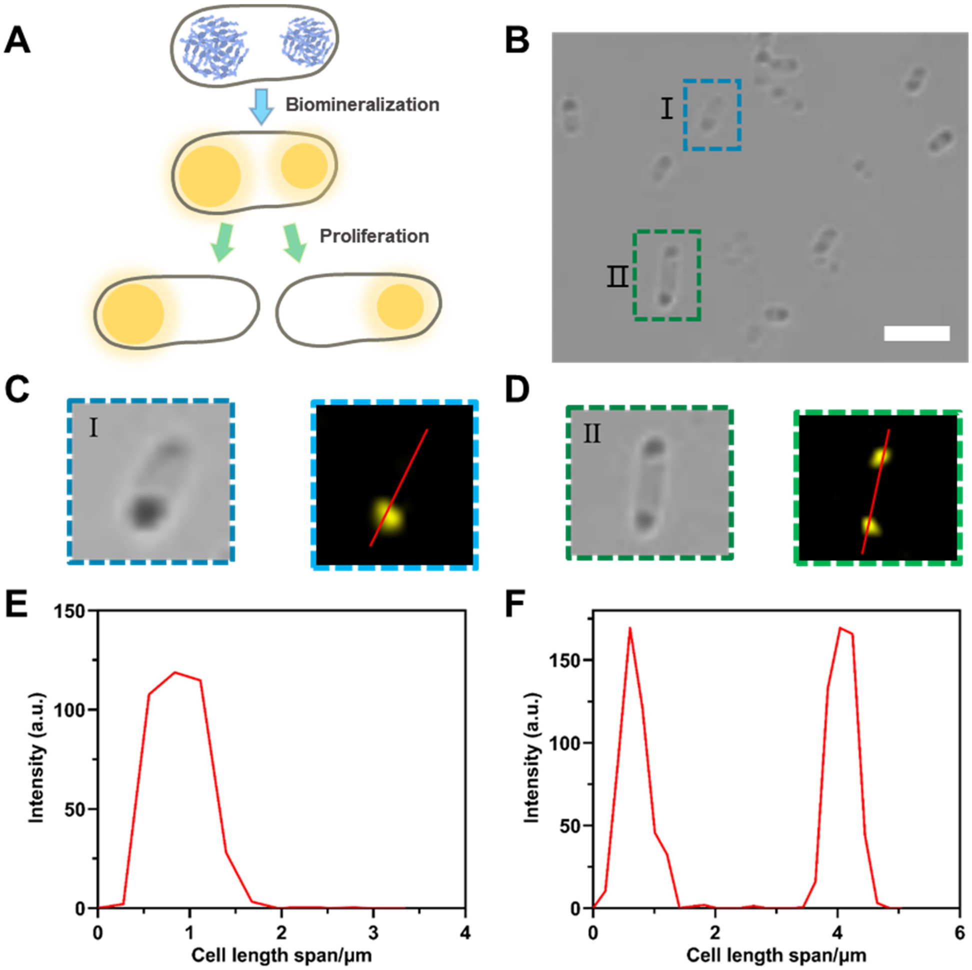

Figure S12. Two different fluorescence distributions appear in the HM/IBSCS. (A) A schematic diagram illustrates that, following induction and ion mineralization, bacteria exhibit two distinct morphologies due to bacterial division. Morphological characteristics of bacteria in the bright field (B, C, D), The bacteria display unipolar (C, E) and bipolar (D, F) fluorescence, respectively.

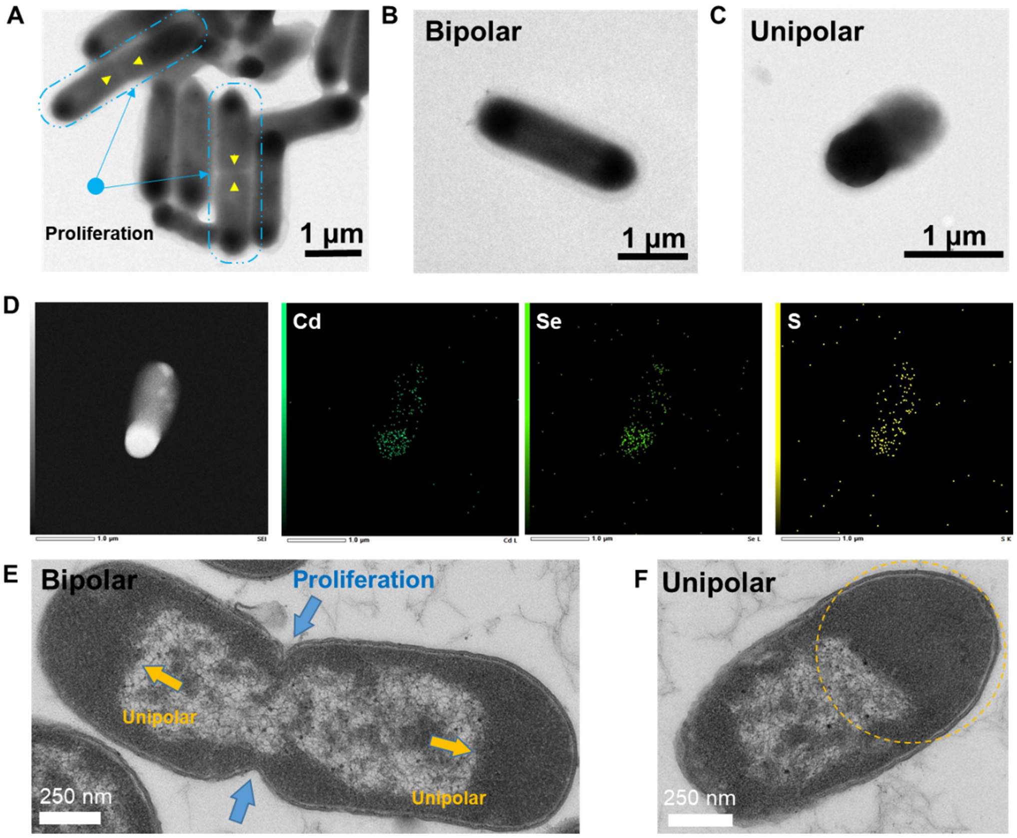

Figure S13. TEM characterizes two different morphologies of *E. coli* present in the HM/IBSCS. (A) TEM images of HM *E. coli* mineralized by CdSexS1-xQDs. Two types of bacteria were observed to exhibit significant adhesion (blue border), with only a unipolar dark region at the extreme of the bacteria, which may be due to bacterial division. (B) The TEM image reveals mineralization of CdSexS1-x QDs at bipolar of the HM/IBSCS. (C) The TEM image demonstrates the unipolar mineralization of CdSexS1-x QDs at bacteria. (D) Cd, Se, and S elements exhibit spatially oriented distribution, with aggregated distribution at the single poles of *E. coli*. (E) Bio-TEM image of thin-sectioned HM/IBSCS, which is undergoing a division process, with a noticeable depression in the middle part of the cell, which is a characteristic of the division process. (F) Bio-TEM image of thin-sectioned HM/IBSCS shows unipolar. Thin-sectioned HM/IBSCS Bio-TEM images display unipolar subcellular compartments.

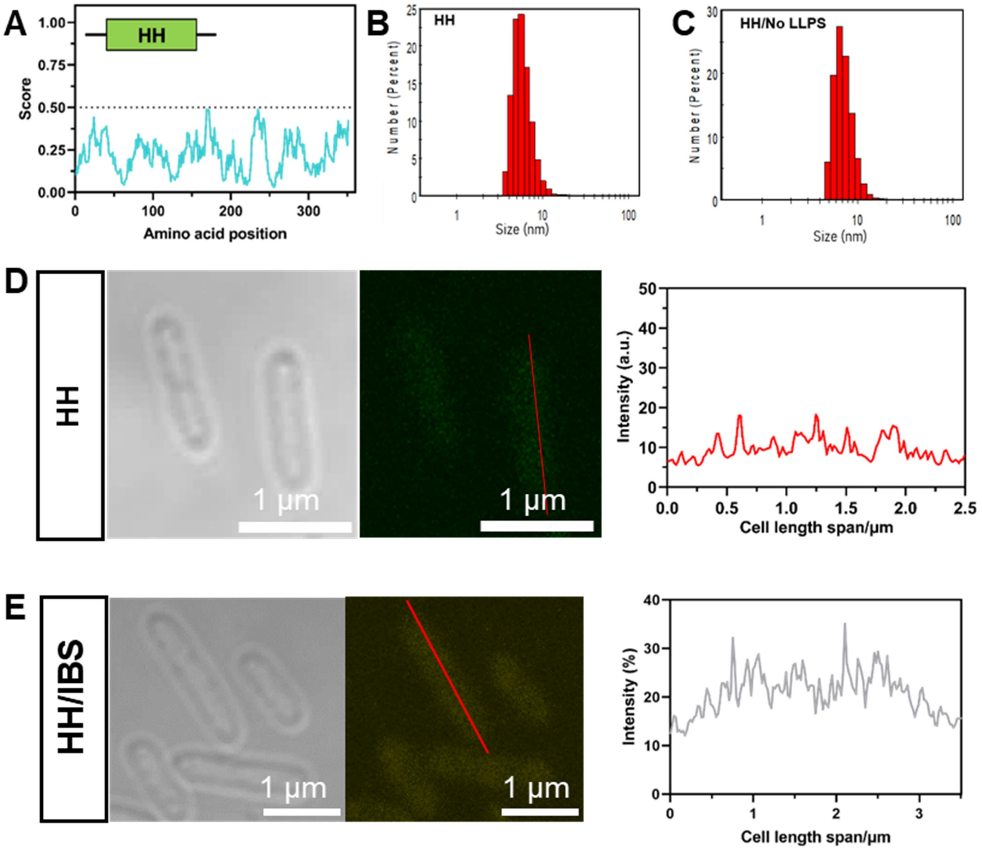

Figure S14. *In vitro* phase separation system based on HH protein. (A) Prediction of protein disorder of hydrogenase fusion with his-tag (HH) using the IUPRed2 program. A score greater than 0.5 (above the horizontal line) indicates an unordered region, while a score less than 0.5 indicates an ordered region. The HH theoretically does not have the ability for LLPS. (B) The particle size of the HH solution did not change before and after mixing (C) with the macromolecular crowding agent dextran 70. (D) The bright-field and confocal laser scanning microscopy images of the *E. coli* expressing empty HH stained with ThT reveal minimal green fluorescence. The fluorescence intensity line-cuts analysis showed no significant change in bacterial fluorescence. (E) The CLSM images for the $CdSe_xS_{1-x}$ QDs mineralized in HH/IBS. In these two biohybrid systems, mineralization of $CdSe_xS_{1-x}$ QDs occurred simultaneously and exhibited consistent fluorescence trends within the cells. Scale bar = 1 μm.

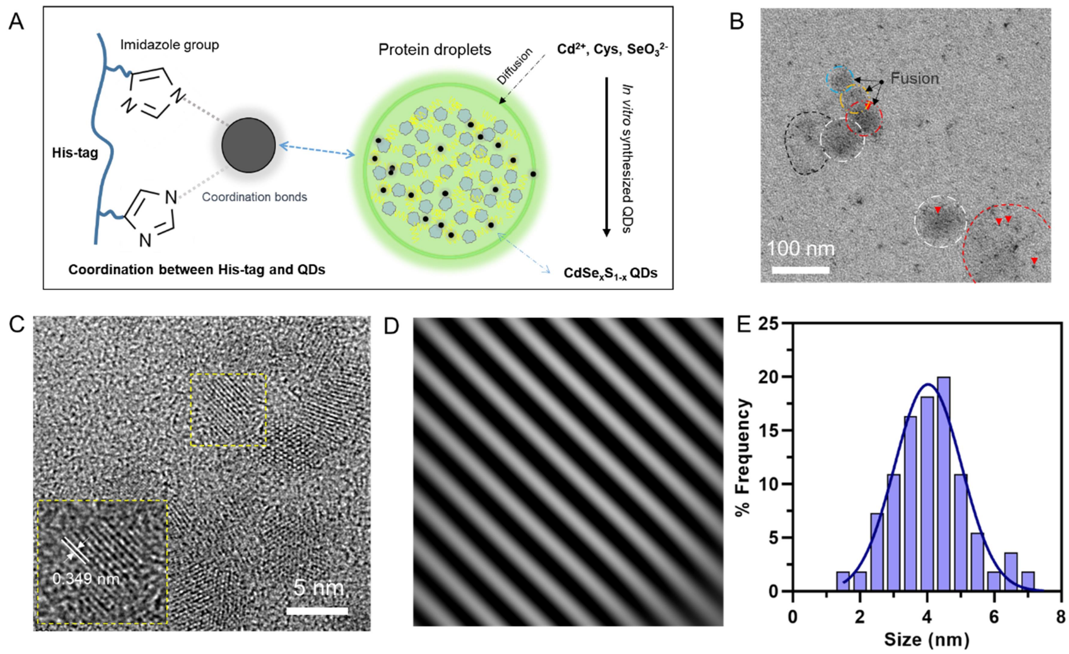

Figure S15. ***In vitro*** **reconstitution of $CdSe_xS_{1-x}$ QDs biosynthesis in HM** protein droplets.

(A) Schematic diagram illustrating the *in vitro* reconstitution of $CdSe_xS_{1-x}$ QDs biosynthesis. In the LLPS protein condensate, the imidazole group of histidine residues in the histidine tag forms a strong coordination bond with $CdSe_xS_{1-X}$ QDs, effectively anchoring the QDs around the hydrogenase. Cys acts as a reducer. (B) In the protein condensate droplets formed in vitro, partial fusion of protein condensates (circular regions) is observed. Additionally, the formation of QDs within the protein condensates is observed (red arrows). (C) The HRTEM images of the $CdSe_xS_{1-X}$ QDs. The inset in the illustration shows an individual nanocrystal with lattice planes spaced at 0.349 nm, scale bar = 5 nm. (D) The related fast Fourier transform (FFT) patterns (inset of C) of the individual nanocrystal $CdSe_xS_{1-x}$QDs. (E) Size distribution of the $CdSe_xS_{1-x}$ QDs synthesized *in vitro*.

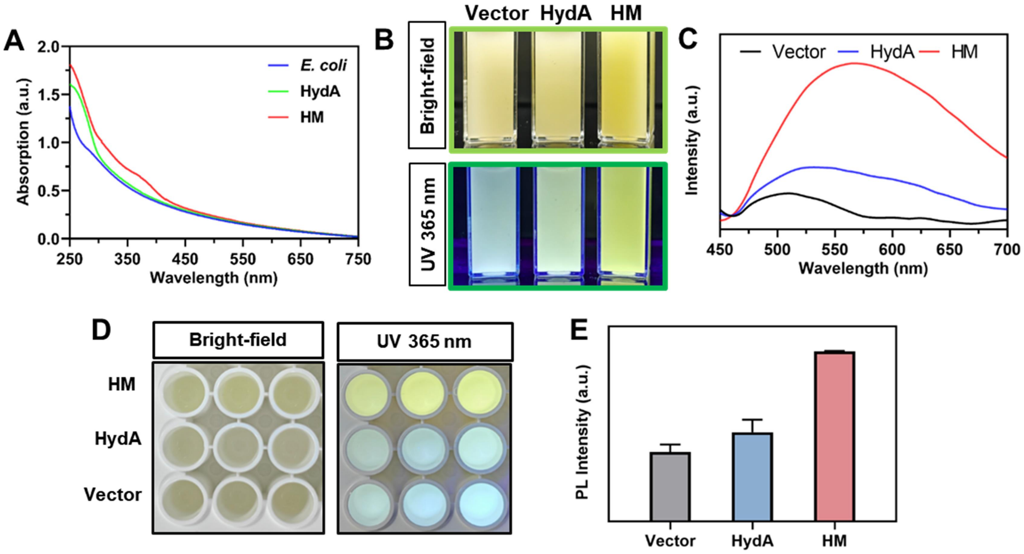

Figure S16. Characterization of QD formation under anaerobic conditions incubation with 1 mM $CdCl_2$ and Na_2SeO_3. (A) UV-vis absorption. The broken *E. coli* cells exhibit a clear absorption peak at 400 nm, which can be attributed to the absorption peak of QDs. (B) The images from the QDs under a bright field or UV 365 nm light irradiation. (C) The fluorescence emission spectrum QDs under excitation at 400 nm shows a maximum emission peak at ~575 nm. (D) Optical image of QDs placed in a 96 well plate under bright field and UV 365 nm irradiation. (E) Measure the fluorescence intensity of lysate *E. coli* cells using a Microplate reader.

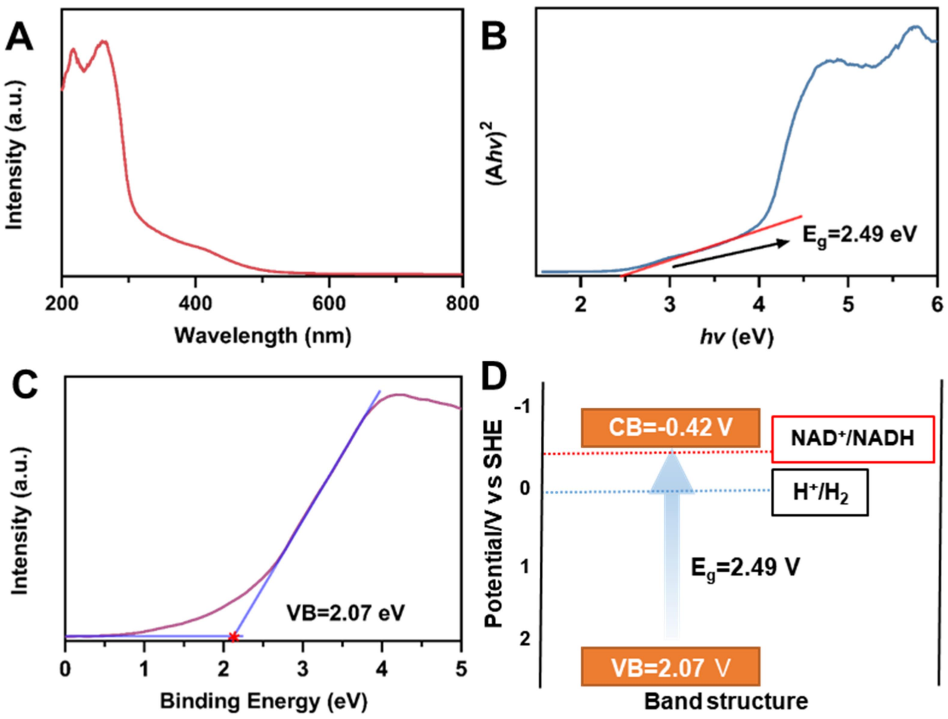

Figure S17. (A) UV-vis-DRS spectra of the QDs. (B) Tauc plot from UV-vis-DRS spectrum, (C) valence band XPS spectra of QDs. (D) a diagram of the band structures of QDs.

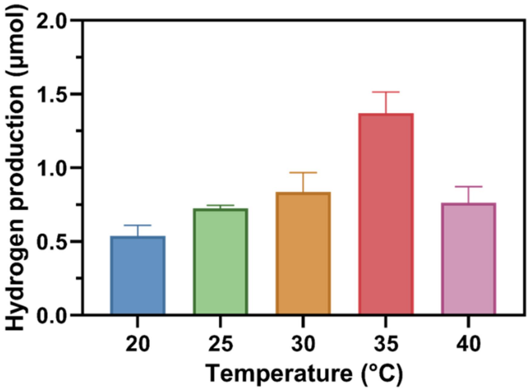

Figure S18. After IPTG induction of hydrogen enzyme expression, the HM strain was transferred to a freshly prepared sterile hydrogen production buffer under anaerobic conditions, and hydrogen production was observed under different temperature conditions (Buffer: 100 mM Tris HCl, 150 mM NaCl, 50 mM Glucose 24 h).

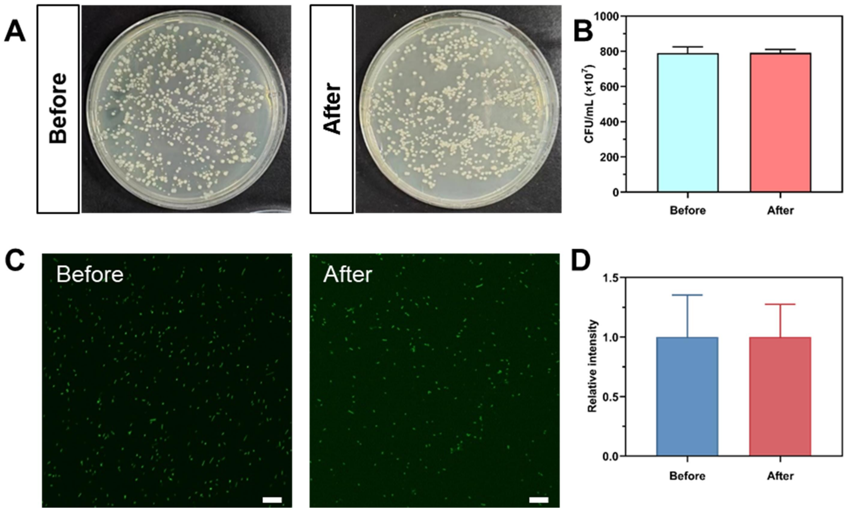

Figure S19. The viability of *E. coli* in the HM/IBSCS. (A) The CFU images and quantitative data (B) of HM/IBSCS after the visible light irradiation. Comparison of ROS content in the HM/IBSCS before and after light irradiation. Fluorescence images of ROS in bacteria before and after light irradiation (C) and the corresponding fluorescence quantification (D). scale bar = 20 μm.

Table S1. Plasmids used in this study

**Plasmids**	**Backbone**	**Description**
pETDuet-1/HydE+HydA-MaSpI8	*pETDuet-1*	the [Fe-Fe]-hydrogenase gene fused to MaSpI8 and Co-expression with HydE, Ampicillin
*pCDFDuet-1/*HydF+HydG	*pCDFDuet-1*	Co-expression of HydF and HydG, Streptomycin
pETDuet-1	*pETDuet-1*	Empty vector, Ampicillin
pCDFDuet-1	*pCDFDuet-1*	Empty vector, Streptomycin
*pETDuet-1/*HydE+HydA	*pETDuet-1*	Co-expression of HydA and HydE, Ampicillin
*pETDuet-1/*HydE+HydA-2×His-tag	*pETDuet-1*	the HydA protein fused to His tag and Co-expression with HydE, Ampicillin

Table S2. Sequences of proteins in this work.

**HydA**	MKTIILNGNEVHTDKDITILELARENNVDIPTLCFLKDCGNFGKCGVC MVEVEGKGFRAACVAKVEDGMVINTESDEVKERIKKRVSMLLDKHE FKCGQCSRRENCEFLKLVIKTKAKASKPFLPEDKDALVDNRSKAIVID RSKCVLCGRCVAACKQHTSTCSIQFIKKDGQRAVGTVDDVCLDDSTC LLCGQCVIACPVAALKEKSHIEKVQEALNDPKKHVIVAMAPSVRTAM GELFKMGYGKDVTGKLYTALRMLGFDKVFDINFGADMTIMEEATEL LGRVKNNGPFPMFTSCCPAWVRLAQNYHPELLDNLSSAKSPQQIFGT ASKTYYPSISGIAPEDVYTVTIMPCNDKKYEADIPFMETNSLRDIDASL TTRELAKMIKDAKIKFADLEDGEVDPAMGTYSGAGAIFGATGGVME AAIRSAKDFAENKELENVDYTEVRGFKGIKEAEVEIAGNKLNVAVIN GASNFFEFMKSGKMNEKQYHFIEVMACPGGCINGGGQPHVNALDRE NVDYRKLRASVLYNQDKNVLSKRKSHDNPAIIKMYDSYFGKPGEGL AHKLLHVKYTKDKNVSKHESA*
**HydE**	MDNIIKLINKAEVTHDLTKDELVTLLKDDTHNEEIYKAADRVREKYV GEEVHLRGLIEFSNICKRNCMYCGLRRDNKNIKRYRLEPDEIIHLAKS AKNYGYQTVVLQSGEDDYYTVEKMKYIVSEIKKLNMAITLSIGEKTF EEYEEYRKSGADRYLIRIETTDKELYEKLDPKMSHENRINCLKNLRKL GYEVGSGCLVGLPNQTIESLADDILFFKEIDADMIGVGPFIPNEDTPLG EEKGGEFFMSVKVTALIRLLLPDINIPATTAMESLYPNGRSIALTSGAN VVMPNVTEGEYRKLYALYPGKICVNDTPGHCRQCISLKINKINRKVSA TKGFRKKSYKESIG*
**HydF**	MDELNSTPKGERLHIALFGKTNVGKSSVINALTSQEIALVSNVKGTTT DPVYKAMELLPLGPVMLIDTAGLDDISDLGELRRGKTLEVLSKTDVAI LVFDVESGITEYDKNIYSLLLEKKIPLIGVLNKIDKKDYKLEDYTSQFK IPIVPISALNNKGINNLKDELIRLAPENDDKFKIVGDLLSPGDIAVLVTPI DKAAPKGRLILPQQQTIRDILESDAIAMVTKEFELRETLDSLRKKPKIV ITDSQVFLKVAADTPKDILMTSFSILMARHKGDLIELARGARAIEDLK DGDKILIAEACTHHRQSDDIGKVKIPRWLRQKTGKKLEFDFSSGFSFP PNIEDYALIVHCAGCMLNRRSMLHRIESSVKKQIPIVNYGVLIAYVQGI LPRALKPFPYADRIFNQSSRN*
**HydG**	MYNVKSKVATEFISDEEIIDSLEYAKQNKSNRELIDSIIEKAKECKGLT HRDAAVLLECDLEDENEKMFKLAREIKQKFYGNRIVMFAPLYLSNYC VNGCVYCPYHHKNKHIARKKLSQEDVKRETIALQDMGHKRLALEA GEDPVNNPIEYILDCIKTIYSIKHKNGAIRRVNVNIAATTVENYKKLKD AGIGTYILFQETYNKKSYEELHPTGPKHDYAYHTEAMDRAMEGGIDD VGIGVLFGLNMYKYDFVGLLMHAEHLEAAMGVGPHTISVPRIRPAD DIDPENFSNAISDEIFEKIVAIIRIAVPYTGMIVSTRESKKTRERVLELGIS QISGGSSTSVGGYVESEPEEDNSSQFEVNDNRTLDEIVNWLLEMNYIP SFCTACYREGRTGDRFMSLVKSGQIANCCQPNALMTLKEYLEDYASS NTQKNGEALIASEVEKIPNEKVKSIVKKHLTELKEGQRDFRF*

**HydA-MaSpI**<br>**8**<br>**(HM)**	MKTIILNGNEVHTDKDITILELARENNVDIPTLCFLKDCGNFGKCGVC<br>MVEVEGKGFRAACVAKVEDGMVINTESDEVKERIKKRVSMLLDKHE<br>FKCGQCSRRENCEFLKLVIKTKAKASKPFLPEDKDALVDNRSKAIVID<br>RSKCVLCGRCVAACKQHTSTCSIQFIKKDGQRAVGTVDDVCLDDSTC<br>LLCGQCVIACPVAALKEKSHIEKVQEALNDPKKHVIVAMAPSVRTAM<br>GELFKMGYGKDVTGKLYTALRMLGFDKVFDINFGADMTIMEEATEL<br>LGRVKNNGPFPMFTSCCPAWVRLAQNYHPELLDNLSSAKSPQQIFGT<br>ASKTYYPSISGIAPEDVYTVTIMPCNDKKYEADIPFMETNSLRDIDASL<br>TTRELAKMIKDAKIKFADLEDGEVDPAMGTYSGAGAIFGATGGVME<br>AAIRSAKDFAENKELENVDYTEVRGFKGIKEAEVEIAGNKLNVAVIN<br>GASNFFEFMKSGKMNEKQYHFIEVMACPGGCINGGGQPHVNALDRE<br>NVDYRKLRASVLYNQDKNVLSKRKSHDNPAIIKMYDSYFGKPGEGL<br>AHKLLHVKYTKDKNVSKHESAWSHPQFEMGSS**HHHHHH**SSGLVPR<br>GSHMASGPGGYGPGQQGPSGPGSAAAAAAAAGPGGYGPGQQTSGP<br>GGYGPGQQGPSGPGSAAAAAAAAGPGGYGPGQQTSGPGGYGPGQQ<br>GPSGPGSAAAAAAAAGPGGYGPGQQTSGPGGYGPGQQGPSGPGSAA<br>AAAAAAGPGGYGPGQQTSGPGGYGPGQQGPSGPGSAAAAAAAAGP<br>GGYGPGQQTSGPGGYGPGQQGPSGPGSAAAAAAAAGPGGYGPGQQ<br>TSGPGGYGPGQQGPSGPGSAAAAAAAAGPGGYGPGQQTSGPGGYGP<br>GQQGPSGPGSAAAAAAAAGPGGYGPGQQTSLE**HHHHHH**GSS*
**HydA-His**<br>**(HH)**	MKTIILNGNEVHTDKDITILELARENNVDIPTLCFLKDCGNFGKCGVC<br>MVEVEGKGFRAACVAKVEDGMVINTESDEVKERIKKRVSMLLDKHE<br>FKCGQCSRRENCEFLKLVIKTKAKASKPFLPEDKDALVDNRSKAIVID<br>RSKCVLCGRCVAACKQHTSTCSIQFIKKDGQRAVGTVDDVCLDDSTC<br>LLCGQCVIACPVAALKEKSHIEKVQEALNDPKKHVIVAMAPSVRTAM<br>GELFKMGYGKDVTGKLYTALRMLGFDKVFDINFGADMTIMEEATEL<br>LGRVKNNGPFPMFTSCCPAWVRLAQNYHPELLDNLSSAKSPQQIFGT<br>ASKTYYPSISGIAPEDVYTVTIMPCNDKKYEADIPFMETNSLRDIDASL<br>TTRELAKMIKDAKIKFADLEDGEVDPAMGTYSGAGAIFGATGGVME<br>AAIRSAKDFAENKELENVDYTEVRGFKGIKEAEVEIAGNKLNVAVIN<br>GASNFFEFMKSGKMNEKQYHFIEVMACPGGCINGGGQPHVNALDRE<br>NVDYRKLRASVLYNQDKNVLSKRKSHDNPAIIKMYDSYFGKPGEGL<br>AHKLLHVKYTKDKNVSKHESAWSHPQFEMGSS**HHHHHH**SSGLVPR<br>GSHMASLE**HHHHHH**GSS*

Yellow highlighted as **MaSpI8/IDPs**

Table S3. Comparison of apparent quantum yields (AQY) of biological hybrid systems constructed under different construction strategies for photocatalytic hydrogen production.

**Photosensitizer@Location**	**Strategy**	**Wavelength (nm)**	**AQE(%)**	**Ref.**
CdS@Extracellular	Biomineralization	470	7.93	1
$CuInS_2$/ZnS@Periplasm	Ingestion	420	6.46	2
$AglnS_2$/In_2S@Extracellular	Biomineralization	720	3.3	3
GaN: ZnO@Extracellular	Physical mixing	420	0.93	4
TiO_2/MV@Extracellular	Physical mixing	420	0.1	5
$CdSe_xS_{1-x}$/Intracellular/ Subcellular compartment	Biomineralization	420	11.63	This work

Table S4. Comparison of the difference in multiple (fold) of the products obtained under the conditions of biological hybridization system and photocatalysis with naked bacteria or photosensitizer.

**Photosensitizer**	**Strain**	**Photosensitizer position**	**Synthesis**	**Efficiency improvement**	**Product**	**Ref.**
TiO_2	*E. coli*	Extracellular	Synthesise	67- fold	H_2	5
TiO_2	*E. coli*	Extracellular	Synthesise	1-fold	H_2	6
Cu_2O/RGO	*S. oneidensis*	Intracellular	Synthesise	46-fold	H_2	7
$CuInS_2$/ZnS	*S. oneidensis*	Periplasmic	Synthesise	8.6-fold	H_2	2
CdS	*E. coli*	Extracellular	Biomineralization	4.5-fold	H_2	8
CdS	*E. coli*	Extracellular	Biomineralization	1.2-fold	H_2	1
CdS	*E. coli*	Extracellular	Biomineralization	3-fold	H_2	9
CdS	*E. coli*	Extracellular	Biomineralization	5-fold	H_2	10
CdS	*E. coli*	Extracellular	Biomineralization	11-fold	H_2	11
$CdSe_xS_{1-x}$	*E. coli*	Intracellular	Biomineralization	3-fold	H_2	12
I-HTCC	*E. coli*	Extracellular	Synthesise	1.5-fold	H_2	13
C3N4	*E. coli*	Intracellular	Synthesise	4-fold	H_2	14
$AglnS_2/In_2S_3$	*E. coli*	Extracellular	Synthesise	1.5-fold	H_2	3
Eosin Y	*S. oneidensis*	Intracellular	Synthesise	10-fold	H_2	15
polymers	*R. palustris*	Extracellular	Synthesise	5-fold	H_2	16
NiCu@CdS	*M. barkeri*	Extracellular	Synthesise	6-fold	CH_4	17
Carbon dot	*M. barkeri*	Extracellular	Synthesise	8-fold	CH_4	18
CdS	*M. barkeri*	Extracellular	Biomineralization	6-fold	CH_4	18
Core-shell QDs	*A. vinelandii*	Extracellular	Synthesise	5-fold	NH_3	19
Au NCs	*A. vinelandii*	Intracellular	Synthesise	8-fold	NH_3	20
InP/ZnSe QDs	*A. vinelandii*	Intracellular	Synthesise	6-fold	NH_3	21
CdS	*S. ovata*	Extracellular	Biomineralization	1-fold	Acetate	22
eosin Y	*E. coli*	Intracellular	Synthesise	10-fold	indanediol	23
Au NCs	*M. thermoacetica*	Intracellular	Synthesise	10-fold	Acetate	24
CdS	*E. coli*	Extracellular	Biomineralization	10-fold	HCOOH	25
Polymer	*Cyanobacteria*	Extracellular	Synthesise	2-fold	O_2	26
$CdSe_xS_{1-x}$	*Engineering E Coli*	Intracellular/ Subcellular compartment	Biomineralization	87-fold	H_2	This work

SI References

(1) Wang, B.,Zeng, C.,Chu, K. H.,Wu, D.,Yip, H. Y.,Ye, L. and Wong, P. K. (2017). Enhanced Biological Hydrogen Production from *Escherichia coli* with Surface Precipitated Cadmium Sulfide Nanoparticles. Adv. Energy Mater. *7*, 1700611.

(2) Luo, B.,Wang, Y.-Z.,Li, D.,Shen, H.,Xu, L.-X.,Fang, Z.,Xia, Z.,Ren, J.,Shi, W. and Yong, Y.-C. (2021). A Periplasmic Photosensitized Biohybrid System for Solar Hydrogen Production. Adv. Energy Mater. *11*, 2100256.

(3) Jiang, Z.,Wang, B.,Yu, J. C.,Wang, J.,An, T.,Zhao, H.,Li, H.,Yuan, S. and Wong, P. K. (2018). $AglnS_2/In_2S_3$ heterostructure sensitization of *Escherichia coli* for sustainable hydrogen production. Nano Energy *46*, 234-240.

(4) Kosem, N.,Honda, Y.,Watanabe, M.,Takagaki, A.,Tehrani, Z. P.,Haydous, F.,Lippert, T. and Ishihara, T. (2020). Photobiocatalytic H_2 evolution of GaN:ZnO and [FeFe]-hydrogenase recombinant E*scherichia coli*. Catalysis Science & Technology *10*, 4042-4052.

(5) Honda, Y.,Hagiwara, H.,Ida, S. and Ishihara, T. (2016). Application to Photocatalytic H_2 Production of a Whole-Cell Reaction by Recombinant *Escherichia coli* Cells Expressing [FeFe]-Hydrogenase and Maturases Genes. Angew. Chem. Int. Ed. *55*, 8045-8048.

(6) Ramprakash, B. and Incharoensakdi, A. (2020). Encapsulated titanium dioxide nanoparticle-Escherichia coli hybrid system improves light driven hydrogen production under aerobic condition. Bioresour. Technol. *318*, 124057.

(7) Shen, H.,Wang, Y.-Z.,Liu, G.,Li, L.,Xia, R.,Luo, B.,Wang, J.,Suo, D.,Shi, W. and Yong, Y.-C. (2020). A Whole-Cell Inorganic-Biohybrid System Integrated by Reduced Graphene Oxide for Boosting Solar Hydrogen Production. ACS Catal. *10*, 13290-13295.

(8) Han, H.-X.,Tian, L.-J.,Liu, D.-F.,Yu, H.-Q.,Sheng, G.-P. and Xiong, Y. (2022). Reversing Electron Transfer Chain for Light-Driven Hydrogen Production in Biotic–Abiotic Hybrid Systems. J. Am. Chem. Soc. *144*, 6434-6441.

(9) Wang, X.-M.,Chen, L.,He, R.-L.,Cui, S.,Li, J.,Fu, X.-Z.,Wu, Q.-Z.,Liu, H.-Q.,Huang, T.-Y. and Li, W.-W. (2022). Anaerobic self-assembly of a regenerable bacteria-quantum dot hybrid for solar hydrogen production. Nanoscale *14*, 8409-8417.

(10) Martins, M.,Toste, C. and Pereira, I. A. C. (2021). Enhanced Light-Driven Hydrogen Production by Self-Photosensitized Biohybrid Systems. Angew. Chem. Int. Ed. *60*, 9055-9062.

(11) Wei, W.,Sun, P.,Li, Z.,Song, K.,Su, W.,Wang, B.,Liu, Y. and Zhao, J. A surface-display biohybrid approach to light-driven hydrogen production in air. Sci. Adv. *4*, eaap9253.

(12) Cui, S.,Tian, L.-J.,Li, J.,Wang, X.-M.,Liu, H.-Q.,Fu, X.-Z.,He, R.-L.,Lam, P. K. S.,Huang, T.-Y. and Li, W.-W. (2022). Light-assisted fermentative hydrogen production in an intimately-coupled inorganic-bio hybrid with self-assembled nanoparticles. Chem. Eng. J. *428*, 131254.

(13) Xiao, K.,Tsang, T. H.,Sun, D.,Liang, J.,Zhao, H.,Jiang, Z.,Wang, B.,Yu, J. C. and Wong, P. K. (2021). Interfacing Iodine-Doped Hydrothermally Carbonized Carbon with *Escherichia coli* through an “Add-on” Mode for Enhanced Light-Driven Hydrogen Production. Adv. Energy Mater. *11*, 2100291.

(14) Wu, D.,Zhang, W.,Fu, B. and Zhang, Z. (2022). Living intracellular inorganic-microorganism biohybrid system for efficient solar hydrogen generation. Joule *6*, 2293-2303.

(15) Honda, Y.,Shinohara, Y. and Fujii, H. (2020). Visible light-driven, external mediator-free H_2 production by a combination of a photosensitizer and a whole-cell biocatalyst: *Escherichia coli* expressing [FeFe]-hydrogenase and maturase genes. Catalysis Science & Technology *10*, 6006-6012.

(16) Wang, Z.,Gao, D.,Geng, H. and Xing, C. (2021). Enhancing hydrogen production by

photobiocatalysis through Rhodopseudomonas palustris coupled with conjugated polymers. J. Mater. Chem. A *9*, 19788-19795.

(17) Ye, J.,Wang, C.,Gao, C.,Fu, T.,Yang, C.,Ren, G.,Lü, J.,Zhou, S. and Xiong, Y. (2022). Solar-driven methanogenesis with ultrahigh selectivity by turning down H_2 production at biotic-abiotic interface. Nat. Commun. *13*, 6612.

(18) Wang, C.,Yu, J.,Ren, G.,Hu, A.,Liu, X.,Chen, Y.,Ye, J.,Zhou, S. and He, Z. (2022). Self-replicating Biophotoelectrochemistry System for Sustainable CO Methanation. Environ. Sci. Technol. *56*, 4587-4596.

(19) Ding, Y.,Bertram, J. R.,Eckert, C.,Bommareddy, R. R.,Patel, R.,Conradie, A.,Bryan, S. and Nagpal, P. (2019). Nanorg Microbial Factories: Light-Driven Renewable Biochemical Synthesis Using Quantum Dot-Bacteria Nanobiohybrids. J. Am. Chem. Soc. *141*, 10272-10282.

(20) Bertram, J. R.,Ding, Y. and Nagpal, P. (2020). Gold nanoclusters cause selective light-driven biochemical catalysis in living nano-biohybrid organisms. Nanoscale Adv. *2*, 2363-2370.

(21) Koh, S.,Choi, Y.,Lee, I.,Kim, G.-M.,Kim, J.,Park, Y.-S.,Lee, S. Y. and Lee, D. C. (2022). Light-Driven Ammonia Production by Azotobacter vinelandii Cultured in Medium Containing Colloidal Quantum Dots. J. Am. Chem. Soc. *144*, 10798-10808.

(22) He, Y.,Wang, S.,Han, X.,Shen, J.,Lu, Y.,Zhao, J.,Shen, C. and Qiao, L. (2022). Photosynthesis of Acetate by *Sporomusa ovata*–CdS Biohybrid System. ACS Appl. Mater. Interfaces *14*, 23364-23374.

(23) Feyza Özgen, F.,Runda, M. E.,Burek, B. O.,Wied, P.,Bloh, J. Z.,Kourist, R. and Schmidt, S. (2020). Artificial Light-Harvesting Complexes Enable Rieske Oxygenase Catalyzed Hydroxylations in Non-Photosynthetic cells. Angew. Chem. Int. Ed. *59*, 3982-3987.

(24) Zhang, H.,Liu, H.,Tian, Z.,Lu, D.,Yu, Y.,Cestellos-Blanco, S.,Sakimoto, K. K. and Yang, P. (2018). Bacteria photosensitized by intracellular gold nanoclusters for solar fuel production. Nat. Nanotechnol. *13*, 900-905.

(25) Wang, X.,Zhang, J.,Li, K.,An, B.,Wang, Y. and Zhong, C. Photocatalyst-mineralized biofilms as living bio-abiotic interfaces for single enzyme to whole-cell photocatalytic applications. Sci. Adv. *8*, eabm7665.

(26) Zeng, Y.,Zhou, X.,Qi, R.,Dai, N.,Fu, X.,Zhao, H.,Peng, K.,Yuan, H.,Huang, Y.,Lv, F., et al. (2021). Photoactive Conjugated Polymer-Based Hybrid Biosystems for Enhancing Cyanobacterial Photosynthesis and Regulating Redox State of Protein. Adv. Funct. Mater. *31*, 2007814.
